# MiR-27a-3p and miR-30b-5p inhibited-vitamin D receptor involved in the progression of tuberculosis

**DOI:** 10.3389/fmicb.2022.1020542

**Published:** 2022-10-11

**Authors:** Min Xiao, Song Yang, An Zhou, Tongxin Li, Jingjing Liu, Yang Chen, Ya Luo, Chunfang Qian, Fuping Yang, Bo Tang, Chunhua Li, Na Su, Jing Li, Mingying Jiang, Shiming Yang, Hui Lin

**Affiliations:** ^1^Department of Gastroenterology, Xinqiao Hospital, Army Medical University, Chongqing, China; ^2^Chongqing Public Health Medical Center, Southwest University, Chongqing, China

**Keywords:** tuberculosis, vitamin D receptor, microRNA, macrophages, differentiation

## Abstract

**Background:**

MicroRNAs (miRNAs) play a vital role in tuberculosis (TB). Vitamin D receptor (VDR), an miRNA target gene, and its ligand, vitamin D_3_ (VitD_3_), have been reported to exert protective effects against TB. However, whether miRNAs can affect the progression of TB by targeting VDR has not been reported.

**Materials and methods:**

Research subjects were selected according to defined inclusion criteria. A clinical database of 360 samples was established, including the subjects’ demographic information, miRNA expression profiles and cellular experimental results. Two candidate miRNAs, miR-27a-3p, and miR-30b-5p, were identified by a high-throughput sequencing screen and validated by qRT–PCR assays. Univariate and multivariate statistical analyses were performed. VDR and NF-kB p65 protein levels were detected by Western blot assays. Proinflammatory cytokine expression levels were detected by enzyme-linked immunosorbent assay (ELISA). Luciferase assays and fluorescence-activated cell sorting (FACS) were further applied to elucidate the detailed mechanisms.

**Results:**

Differential miRNA expression profiles were obtained, and miR-27a-3p and miR-30b-5p were highly expressed in patients with TB. These results showed that the two miRNAs were able to induce M1 macrophage differentiation and inhibit M2 macrophage differentiation. Further experiments showed that the two miRNAs decreased the VDR protein level and increased proinflammatory cytokine secretion by macrophages. Mechanistically, the miRNAs targeted the 3′ untranslated region (3′UTR) of the VDR mRNA and thereby downregulated VDR protein levels by post-transcriptional regulation. Then, due to the reduction in VDR protein levels, the NF-kB inflammatory cytokine signaling pathway was activated, thus promoting the progression of TB.

**Conclusion:**

Our study not only identified differentially expressed miRNAs between the TB and control groups but also revealed that miR-27a-3p and miR-30b-5p regulate proinflammatory cytokine secretion and macrophage differentiation through VDR in macrophages. Thus, these two miRNAs influence the progression of TB.

## Background

Tuberculosis (TB) is one of the top 10 causes of death and the leading cause attributable to a single infectious agent (*Mycobacterium tuberculosis*), ranking above HIV/AIDS. Approximately 1.7 billion people have contracted and been infected with *M. tuberculosis* worldwide, and 1.7 million people die from TB each year (Daley, 2019; Sinha and Hochberg, 2019; World Health Organization [WHO], 2019). *Mycobacterium tuberculosis*, the pathogen that causes TB, is an intracellular parasitic bacterium that infects humans. *M. tuberculosis* can inhibit the host immune response and escape immune surveillance (Goldberg et al., 2014). It is difficult to completely remove *M. tuberculosis* from the host, and once a host becomes infected, *M. tuberculosis* mostly causes latent infection and has a symbiotic relationship with the host (Jagielski et al., 2016). There are many theories about the mechanism underlying the intracellular pathogenesis of *M. tuberculosis*. However, these theories have not yet led to effective anti-TB treatments (Pieters, 2008; Jiang et al., 2018). The diagnosis of TB also needs to be improved and supplemented (McNerney et al., 2012). For these reasons, further research and exploration of the diagnosis and treatment of TB are still urgently needed.

MicroRNAs (MiRNAs) play vital roles in promoting the progression of many diseases (Bertoli et al., 2015; Rupaimoole and Slack, 2017; Dai et al., 2022; Ren et al., 2022). MiRNAs can cleave or repress the mRNAs of target genes through the RNA-induced silencing complex (RISC) (Bartel, 2004; Mohr and Mott, 2015). Studies have reported that miR-155 expression is enriched in active TB (Etna et al., 2018). MiR-29a, miR-21, miR-99b, miR-652, and miR-146 were identified as potential novel biomarkers of TB and could be used to predict responses to treatment (Barry et al., 2018). The high expression of certain miRNAs in TB suggests that some miRNAs are related to the progression of TB.

Vitamin D_3_ (VitD_3_), a steroid hormone, is thought to exert anti-inflammatory effects and play a vital function in innate immunity against intracellular pathogens (Wallis and Zumla, 2016). Studies have reported that VitD_3_ plays an important role in innate immunity against TB (Bekele et al., 2018; Jimenez-Sousa et al., 2018; Ayelign et al., 2020). VitD_3_ exerts its biological effects by binding to the vitamin D receptor (VDR) complex (Heikkinen et al., 2011; Cui et al., 2018), which activates and regulates multiple cellular pathways (Liu et al., 2006; White, 2012). It has been reported that the VitD_3_ levels in patients with active TB are lower than those in control individuals (Nnoaham and Clarke, 2008). Studies have shown that VitD_3_ is a protective factor in TB (Baeke et al., 2010). Studies have also reported that VDR, a receptor of VitD_3_, plays a vital role in TB. A decrease in VDR protein levels causes defects in VDR signaling, which impairs immunity against TB (Selvaraj et al., 2009). Polymorphisms in VDR, such as *Apa*I, *Bsm*I, *Fok*I, and *Taq*I, might affect susceptibility to TB (Joshi et al., 2014; Wu et al., 2015). It has been reported that miR-1204 is able to target VDR in breast cancer, leading to a poor prognosis (Liu X. et al., 2018). MiR-125a can target VDR to promote the occurrence and progression of liver fibrosis (He et al., 2021). Therefore, we wanted to explore whether any particular miRNAs are involved in TB regulation and are likely to depress TB by targeting VDR.

**FIGURE 1:**
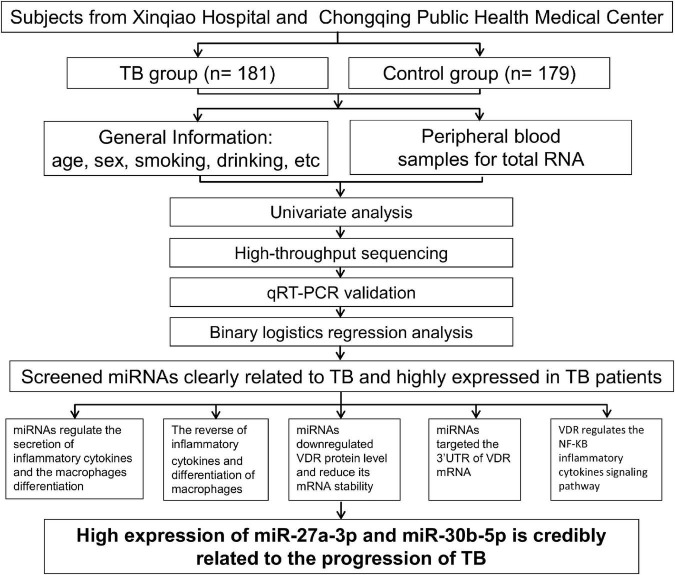
Flow chart. The summary of the current study’s logical flow.

To determine whether miRNA expression and VDR protein levels are correlated in TB, we studied these factors in a TB group and a control group. Subjects came from two medical institutions in Chongqing, and 181 TB patients and 179 control individuals were selected according to the inclusion criteria. A case report form including gender, living situation, education, cigarette smoking, body mass index (BMI), hypertension, etc., was used to obtain demographic information from the subjects. Peripheral venous blood samples collected from subjects were used for high-throughput sequencing and qRT–PCR validation. After univariate analysis and data processing, logistic regression analysis was performed, and two TB-associated miRNAs were screened. Finally, the two miRNAs were studied both phenotypically and mechanistically in monocytes by cytological experiments (Figure 1).

## Materials and methods

### Case-control study

Human subjects were recruited for this study mainly from Xinqiao Hospital and Chongqing Public Health Medical Center between June 1, 2019, and December 31, 2019. Demographic information, clinical information, and peripheral blood specimens were collected from 360 subjects. Approximately 181 subjects were recruited into the case group, and 179 subjects were recruited into the control group, excluding previous exposure to TB.

The patients were selected according to the following criteria: (1) patient was older than 18 years of age, of either sex; (2) patient was Han Chinese with a family that had lived in Chongqing for more than two generations; (3) patient had poisoning symptoms such as sputum, hemoptysis and emaciation, fatigue, night sweating, or low fever; (4) patient had at least one radiographic examination (X-ray and CT) suggesting pulmonary TB lesions; (5) patient had positive results on TB diagnostic tests such as sputum smear, sputum culture or blood molecular biology; (6) patient had Swiss cheese lesions or granuloma formation by pathological biopsy; and (7) patient was diagnosed with secondary pulmonary TB based on the symptoms, radiographic examination, etiological and pathological findings, regardless of whether they affected a single lung or both lungs and regardless of the presence of extrapulmonary TB.

Patients were excluded according to the following criteria: (1) patient had consanguineous parents; (2) patient had other severe diseases (cancer, immune deficiency disease, pulmonary abscess, etc.); or (3) patient had latent TB.

The control individuals were selected according to the following inclusion and exclusion criteria: (1) Individual who was older than 18 years of age, of either sex was eligible; (2) Individual who was Han Chinese with a family that had lived in Chongqing for more than two generations was eligible; (3) Individual without active or latent TB identified by clinical manifestations, radiographic examination and Purified Protein Derivative (PPD) tests was eligible. (4) Who with a history of TB exposure was excluded; or (5) Other exclusion criteria were the same as the TB group.

Additionally, we collected clinical samples of peripheral venous blood for experimental studies, and from these samples, we isolated peripheral blood mononuclear cells (PBMCs) and plasma following appropriate experimental methods for subsequent research. All the data are available, and our research has been approved by the Xinqiao Hospital Ethics Committee.

### Peripheral blood mononuclear cells and plasma specimens

Peripheral venous blood was collected, treated with the anticoagulation agent ethylenediaminetetraacetic acid (EDTA), and centrifuged to isolate blood cells and plasma. PBMCs in blood were separated by using density gradient centrifugation (Böyum and Scand, 1968; Harris and Ukayiofo, 1969). Blood was added down the wall of a tube containing human lymphocyte separation medium (Dakewe Co., Beijing, China, 711101X), and the PBMCs in the blood were purified by density gradient centrifugation (800 × *g*, 30 min). Plasma and PBMC specimens were stored at −80°C and in liquid nitrogen, respectively. The PBMCs used for RNA sequencing and miRNA/mRNA qRT–PCR were obtained from both TB patients and control individuals. PBMCs used for other cytological experiments were obtained from volunteers in the lab. Plasma specimens were used to measure concentration of 1, 25 (OH)_2_ D_3_ by enzyme-linked immunosorbent assay (ELISA) assay.

### RNA sequencing

Following the manufacturer’s protocol for RNAiso Plus reagent (Takara Bio, Osaka, Japan), we extracted total RNA from the PBMCs of control individuals and TB patients. To obtain the miRNA differential expression profiles between TB patients and control individuals, the PBMC samples of five TB patients and five control individuals were selected to match the age and sex of the subjects. After total RNA extraction from PBMCs, we reverse transcribed the RNA into cDNA with the PolyA RT–PCR method. The cDNA was amplified by qPCR and then purified by PAGE. The differential miRNA expression profile was obtained by using high-throughput sequencing technology (LC-bio Technologies Co., Ltd., Hangzhou, China).

### Reverse transcription and miRNA/mRNA qRT–PCR

Total RNA was extracted with RNAiso Plus reagent (TaKaRa Bio, T9109). PrimeScript RT reagent kit with gDNA eraser (TaKaRa Bio, RR047A), SYBR qRT–PCR (TaKaRa Bio R4130-03) and the MiR-X miRNA First-Strand Synthesis Kit were used to reverse transcribe cDNA from mRNA and miRNA, respectively. The cDNA was analyzed by a qPCR kit (TaKaRa Bio RR820A) and GoTaq qPCR Master Mix (Promega, A6001, Madison, WI, United States) with a QuantStudio 3 and ViiATM7 quantitative real-time PCR instrument (Applied Biosystems, Waltham, MA, United States, with technical support by BioWavelet Co., Ltd., Chongqing, China). Gene-specific primers, oligo-dTs and random primers for the reverse transcription of miRNAs were synthesized by GeneCopoeia (Guangzhou, China) and Sangon Biotech (Shanghai, China).

### Cell culture and reagents

We purchased the THP-1 and U-937 cell lines from the Shanghai Institute for Biological Sciences (Shanghai, China). The HEK-293T cell line was ordered from the National Infrastructure of Cell Line Resource (Beijing, China). We genotyped all cell lines and tested them for mycoplasma contamination though Shanghai Biowing Applied Biotechnology Co., Ltd. (Shanghai, China) Primary PBMCs were purified from the peripheral venous blood of the subjects by density gradient centrifugation as described above. We used DMEM and RPMI 1640 medium (HyClone, Logan, UT, United States) to culture HEK-293T cells and monocytes (THP-1, U-937, and PBMCs), respectively. We supplemented the culture media with 10% FBS (HyClone, Logan, UT, United States), 0.1 mg/ml streptomycin and 100 U/ml penicillin (Beyotime, Beijing, China). We cultured cells in an incubator (Thermo Fisher, Waltham, MA, United States) at 37°C, 1 atm and 5% CO_2_.

### Culture medium and plasma analysis by enzyme-linked immunosorbent assay

The proinflammatory cytokine levels in the culture medium and concentration of 1, 25 (OH)_2_ D_3_ in plasma samples were measured by ELISA. The ELISA kits were purchased from 4A BIOTECH company (Beijing, China), and the three proinflammatory cytokines that were assessed were interleukin-1 beta (IL-1β) (CHE0001, 96t), interleukin-6 (IL-6) (CHE0009, 96t), and tumor necrosis factor alpha (TNF-α) (CHE0019, 96t). The minimum concentration that could be detected was 7 pg/ml for all three kits. Centrifugation was performed at 800 rpm/min for 5 min to remove cells and obtain culture medium. Peripheral venous blood was treated with the anticoagulant EDTA and centrifuged (1100 rpm, 15 min) to obtain plasma. Cell culture medium and plasma were detected by ELISA. The reference range of 1, 25 (OH)_2_ D_3_ ELISA kit (EHC9044) is 15–60 ng/ml. All the experiments were performed in accordance with protocols of reagent kits. All experiments were repeated at least two times in triplicate.

### Plasmid, microRNA mimic and microRNA inhibitor transfection

The VDR coding sequence (CDS) was subcloned into pCDNA3.1 (+) by Sangon Biotech. We purchased pmirGLO dual luciferase plasmids carrying the VDR 3′ untranslated region (3′UTR) from YouBio Biological Company (Changsha, China). We named these plasmids WT, ΔWT1, ΔWT2, ΔWT3, ΔWT4, ΔWT5, ΔWT6, ΔWT7, and ΔWT8 and the corresponding mutant plasmids MUT1, MUT2, ΔMUT4, ΔMUT6, and ΔMUT8. We synthesized the miRNA mimics and inhibitors by GeneCopoeia Company (Guangzhou, China) and RiboBio (Guangzhou, China). We transfected the pmirGLO plasmids, miRNA mimics, and inhibitors into cells with Lipofectamine 3000 (Thermo Fisher, Waltham, MA, United States) and analyzed the cells at 48 h post-transfection. MiRNA mimics with Sulfo-Cyanine5 (Cy-5) dye were transfected into monocytes, and the transfection efficiency was observed by fluorescence microscopy (OLYMPUS IX83, UIS2 optical system). All experiments were repeated at least three times.

### Protein extraction and western blot

We harvested and lysed cells with 5 × loading buffer (Beyotime, Beijing, China) after culture for 48 h. We fully lysed the cell lysates with a vortex mixer (Thermo Fisher, Waltham, MA, United States) at 3000 rpm. The protein samples were then incubated in a dry bath incubator (Thermo Fisher, Waltham, MA, United States) at 100°C for 10 min to denature the proteins. We subjected protein samples to SDS–PAGE for gel electrophoresis, transferred the gel to PVDF membranes (Millipore, Boston, MA, United States) for transfer electrophoresis, and then exposed them by ECL (Thermo Fisher, Waltham, MA, United States). We purchased primary antibodies against VDR (D2K6W) and GAPDH (D4C6R) from Cell Signaling Technology (CST, Boston, MA, United States). We obtained HRP-conjugated antibody (ZB-2301, ZB-2305) from ZSGB-Bio Company (Beijing, China). All experiments were repeated at least three times.

### MicroRNA post-transcriptional regulation

We transfected the mimics into cells with the Lipofectamine 3000 reagent. The cells were cultivated for 36 h, and then the transcription inhibitor actinomycin D (Act D) (CST, Boston, MA, United States) was added to the cell cultures. Subsequently, we measured VDR mRNA expression by qRT–PCR in the treatment and control groups at 0, 3, 6, and 9 h. Additionally, we measured the expression levels of miR-27a and miR-30b-5p to assess the transfection efficiency. All experiments were repeated at least three times.

### Cell differentiation and flow cytometry assay

Monocytes were differentiated into different macrophage subsets by stimulation with different cytokines. THP-1 and U-937 cells were induced to polarize into M0 macrophages after stimulation by phorbol 12-myristate 13-acetate (PMA) (100 nM) 24 h later. Then, they were polarized to M1 macrophages after stimulation with lipopolysaccharide (LPS) (100 nM) and interferon-gamma (IFN-γ) (20 nM) or polarized to M1 macrophages after stimulation with interleukin-4 (IL-4) (20 nM) and interleukin-13 (IL13) (20 nM) 48 h later. PBMCs were first induced to differentiate into M0 macrophages by stimulation with human colony stimulating factor (h-CSF) (20 nM) for 5 days. Then, they were polarized into M1 or M2 macrophages as described above. Macrophage polarization was confirmed by flow cytometric analysis. We used cell surface markers to identify M0 (CD11b and CD68), M1 (CD40, CD64, CD86, etc.) and M2 (CD163, CD206, CD180, etc.) macrophages. After polarization was completed, we washed the cells once with PBS, scraped them gently and transferred them into fluorescence-activated cell sorting (FACS) tubes. We used fluorochrome-tagged monoclonal antibodies (Dakewe Co., Beijing, China) to stain the cells. CD11b (Pacific Blue) was used to identify M0 macrophages, CD64 (FITC) was used to identify M1 macrophages, and CD206 (PE) and CD163 (CY 7) were used to identify M2 macrophages. After labeling, we washed and resuspended the cells in PBS at least two times, and we analyzed the cells with FlowJo v10.5.3 by a Gallios flow cytometer (Beckman Coulter, Pasadena, CA, United States). All experiments were repeated at least three times.

### Luciferase reporter assay

After reaching 50% confluence, HEK-293T cells were prepared for exogenous nucleic acid transfection. The pmirGLO-VDR-3′UTR plasmids (WT, ΔWT1, ΔWT2, ΔWT3, ΔWT4, ΔWT5, ΔWT6, ΔWT7, ΔWT8, MUT1, MUT2, ΔMUT4, ΔMUT6, and ΔMUT8) were transfected into HEK-293T cells, and either 200 nM miRNA mimics or 200 nM miRNA inhibitors were also transfected at the same time. Forty-eight hours later, we measured the luciferase activity. All transfection experiments we performed were repeated at least three times in triplicate, and the luciferase activity data were normalized.

### Statistical analysis

Continuous variable data are displayed as the mean ± SD, and categorical variable data are displayed as percentages according to the case group and control group. Statistical analyses were performed with SPSS 26.0 software, and VDR and miRNA expression levels were analyzed by GraphPad Prism 8.0. Univariate difference analysis was applied to all factors between the case group and control group. Logistic regression analysis was applied to the association between miRNA expression and other factors in the 360 subjects. Logistic regression and Pearson’s rank correlation test were used to calculate the correlation coefficients. For comparisons, the Mann–Whitney *U*-test was used if no significantly different variances existed between the two groups. To calculate the *p*-value, the unpaired *t*-test analysis was performed.

## Results

### A case–control study shows that univariate factors are clearly associated with tuberculosis

We collected general information about the subjects, such as age, sex, residence, and education, and analyzed this information by using univariate statistics (Table 1). Based on this basic information, there were significant differences in sex and age between the case and control groups. The TB group had a greater proportion of males and younger ages than the control group. Patients from rural areas and with low incomes and lower education levels accounted for a larger proportion of the case group. In addition, habits such as smoking and drinking also differed significantly between the case and control groups. Smoking and drinking behaviors seemed to increase susceptibility to TB. Poor nutrition, as indicated by a lower BMI, was more common in the TB group. These results are consistent with those of a previous report (Panda et al., 2019). We also found that diabetes was more common in the TB group than in the control group. Altogether, these results indicated that TB is a disease associated with many univariate factors.

**TABLE 1:** General information of the case control study (*N* = 360).

Univariate factor	Statistic	Total (*n* = 360)	Control (*n* = 179)	TB (*n* = 181)	*X*^2^/*t*/ *Z*-value	*P*-value
Gender (*n*, %)	Male	238 (66.11)	103 (57.54)	135 (74.59)	11.669	0.001
	Female	122 (33.89)	76 (42.46)	46 (25.41)		
Age	Mean ± sd	50.51 ± 15.15	53.44 ± 12.41	47.61 ± 16.98	3.72	<0.001
Habitat (*n*, %)	Town	172 (47.78)	120 (67.04)	52 (28.73)	52.938	<0.001
	Rural area	188 (52.22)	59 (32.96)	129 (71.27)		
Education (*n*, %)	Primary school	129 (35.83)	55 (30.73)	74 (40.88)	26.344	<0.001
	Junior high school	65 (18.06)	51 (28.49)	14 (7.73)		
	Senior high school	84 (23.33)	36 (20.11)	48 (26.52)		
	College	82 (22.78)	37 (20.67)	45 (24.86)		
Income (10,000¥/year)	Mean ± sd	4.73 ± 3.29	5.32 ± 3.75	4.15 ± 2.65	3.406	0.001
Cigarette (*n*, %)	Smoking	123 (34.17)	35 (19.55)	88 (48.62)	31.186	0.001
	No smoking	197 (54.72)	125 (69.83)	72 (39.78)		
	Quit smoking	40 (11.11)	19 (10.61)	21 (11.6)		
Alcohol (*n*, %)	Drinking	102 (28.33)	36 (20.11)	66 (36.46)	12.645	0.002
	No drinking	221 (61.39)	125 (69.83)	96 (53.04)		
	Quit drinking	37 (10.28)	18 (10.06)	19 (10.5)		
BMI	Mean ± sd	21.67 ± 3.5	23.27 ± 3.05	20.09 ± 3.2	9.642	<0.001
Hypertension (*n*, %)	No	335 (93.06)	168 (93.85)	167 (92.27)	0.352	0.553
	Yes	25 (6.94)	11 (6.15)	14 (7.73)		
Diabetes (*n*, %)	No	335 (93.06)	175 (97.77)	160 (88.4)	12.221	<0.001
	Yes	25 (6.94)	11 (6.15)	14 (7.73)		
Coronary heart disease (*n*, %)	No	353 (98.06)	174 (97.21)	179 (98.9)	1.345	0.246
	Yes	7 (1.94)	5 (2.79)	2 (1.1)		

We counted basic and clinic information for human subjects and performed a univariate analysis of each factor. Among them, gender, age, habitat, education, income, smoking, drinking, and diabetes were significantly different in the Tuberculosis group. And lower body mass index (BMI) was more common in TB group.

### MiR-27a-3p and miR-30b-5p are highly expressed in the tuberculosis group

**FIGURE 2:**
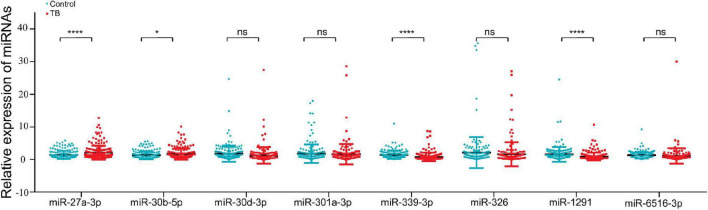
MiR-27a-3p and miR-30b-5p expression was higher in the tuberculosis group than in the control group. The qRT–PCR was used to validate the eight upregulated miRNAs (181 TB patients, 179 control individuals). MiR-27a-3p and miR-30b-5p were highly expressed in the TB group. The expression of four miRNAs (miR-30d-3p, miR-301-3p, miR-326, and miR-6516-3p) was not different between the TB group and the control group, and the remaining two miRNAs (miR-339-3p and miR-1291) were low expressed in the TB group. *P < 0.05, **P < 0.01, ***P < 0.001, ****P < 0.0001, ns = not significant.

We tried to determine whether any miRNAs that might influence TB progression were expressed at higher levels in the TB group than in the control group. MiRNA expression was measured in two steps. First, we performed high-throughput sequencing to determine the differential miRNA expression profile between the two groups (GES207224). Second, qRT–PCR was used in a case–control study to verify the miRNAs that were identified as being highly expressed in the differential expression profiles. These studies revealed that 8 out of 47 differentially expressed miRNAs were upregulated and 15 were downregulated in the TB patient group compared with the control group (Supplementary Figures 1A–C). We verified the expression of these eight miRNAs in 179 control individuals and 181 TB patients, and the results indicated that miR-27a-3p and miR-30b-5p had significantly higher expression in the TB group (Figure 2).

### MiR-27a-3p and miR-30b-5p have a significant association with tuberculosis after adjusting for confounders

To determine whether the associations of miR-27a-3p and miR-30b-5p with TB confounded the effects of the other univariate factors, we corrected the other univariate factors. These two miRNAs, together with previously identified univariate factors related to TB, were analyzed by binary logistic regression (Table 2) and corrected with Model 1, Model 2, and Model 3. Multivariate statistical analysis revealed that after adjusting for the univariate factors in Model 1, Model 2, and Model 3, the two miRNAs were still closely associated with and highly expressed in the TB group. The results indicated that miR-27a-3p and miR-30b-5p have a significant association with TB.

**TABLE 2:** Correlation between miR-27a-3p, miR-30b-5p and tuberculosis (Binary logistics regression).

miRNA	Model	*B*-value	SEM	Wald	*P*-value	OR	OR	
							
							Lower limit	Upper limit
hsa-miR-30b-5p	Uncorrected	0.184	0.079	5.47	0.019	1.202	1.03	1.403
	Model 1	0.257	0.095	7.359	0.007	1.293	1.074	1.556
	Model 2	0.231	0.097	5.71	0.017	1.26	1.042	1.523
	Model 3	0.209	0.101	4.306	0.038	1.233	1.012	1.502
hsa-miR-27a-3p	Uncorrected	0.258	0.073	12.626	0.001	1.294	1.123	1.492
	Model 1	0.32	0.089	12.931	0.001	1.377	1.157	1.64
	Model 2	0.297	0.092	10.439	0.001	1.346	1.124	1.612
	Model 3	0.279	0.097	8.258	0.004	1.321	1.093	1.598

Model 1: Correction factors: gender, age, BMI. Model 2: Model 1 correction factors + cigarette, alcohol. Model 3: Model 1 + Model 2 correction factors + hypertension, diabetes, coronary heart disease.

### MiR-27a-3p and miR-30b-5p promote proinflammatory cytokine expression and the differentiation of M1 macrophages

**FIGURE 3:**
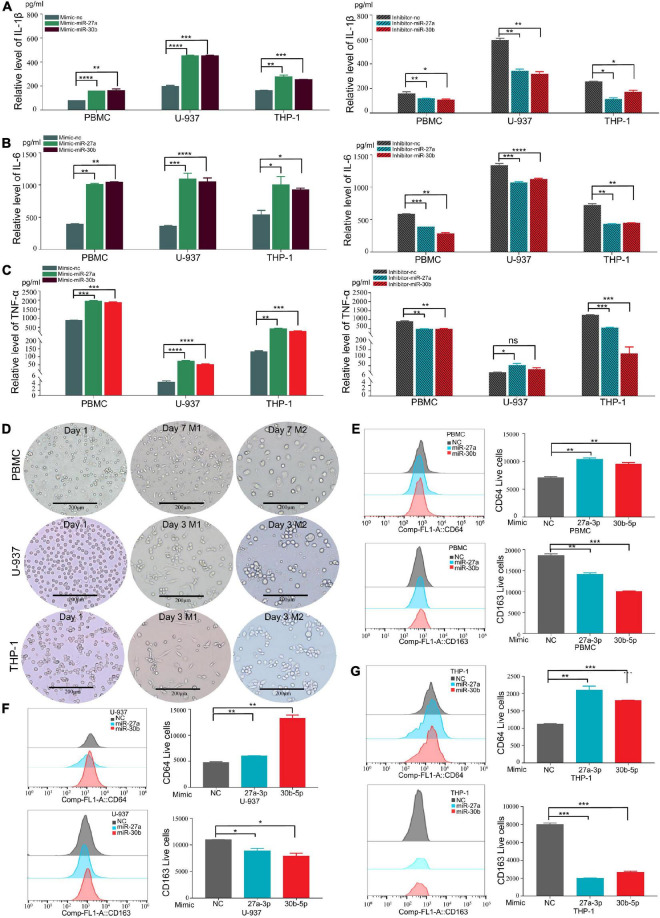
MiR-27a-3p and miR-30b-5p promote proinflammatory cytokine expression and induce monocytes differentiation into M1 macrophages while inhibiting monocytes differentiation into M2 macrophages. **(A)** The expression of IL-1β in monocytes (THP-1, U-937, and PBMCs) when miR-27a-3p and miR-30b-5p were overexpressed or knocked down. Changes in the levels of inflammatory cytokines in culture media were measured by ELISA 48 h later. When miR-27a-3p and miR-30b-5p were overexpressed, IL-1β cytokine secretion was significantly increased in the treatment group compared with the control group. IL-1β cytokine secretion was obviously decreased in the treatment group compared with the control group when the miRNAs were knocked down. **(B)** The expression of IL-6 in monocytes significantly increased when miR-27a-3p and miR-30b-5p were overexpressed. IL-6 cytokine secretion was decreased when the miRNAs were knocked down. **(C)** The expression of TNF-α in monocytes significantly increased when miR-27a-3p and miR-30b-5p were overexpressed. TNF-α cytokine secretion was decreased when the miRNAs were knocked down. **(D)** Phorbol 12-myristate 13-acetate (PMA) (100 nM) was added to THP-1 and U-937 culture medium, and human colony-stimulating factor (h-CSF) (20 nM) was added to PBMCs culture medium. THP-1 and U-937 had differentiated into M0 macrophages 24 h later, and PBMCs had differentiated into M0 macrophages 5 days later. Then, lipopolysaccharide (LPS) (100 nM) and interferon-gamma (IFN-γ) (20 nM) were added to induce differentiation into M1 macrophages, while interleukin-4 (IL-4) (20 nM) and interleukin-13 (IL13) (20 nM) were added to induce differentiation into M2 macrophages. After 48 h, the shapes of cells gradually changed, It showed different types of M1 and M2 macrophages. **(E)** Compared with the negative control, miR-27a-3p and miR-30b-5p could significantly induce PBMCs differentiation into M1 macrophages (CD64). Conversely, miR-27a-3p and miR-30b-5p significantly inhibited PBMCs differentiation into M2 macrophages (CD163). **(F)** miR-27a-3p and miR-30b-5p also significantly induced U-937 differentiation into M1 macrophages (CD64) and similarly inhibited U-937 differentiation into M2 macrophages (CD163). **(G)** The same phenotypes were also validated in THP-1. *P < 0.05, **P < 0.01, ***P < 0.001, ****P < 0.0001, ns = not significant.

Proinflammatory cytokines (IL-1β, IL-6, and TNF-α) were reported to play crucial roles in promoting the progression of TB (Nair et al., 2009; Gong et al., 2019; Ravan et al., 2019). To determine whether miR-27a-3p and miR-30b-5p are related to TB progression by regulating the secretion of proinflammatory cytokines, we transfected miR-27a-3p and miR-30b-5p mimics and inhibitors into cells and measured proinflammatory cytokine secretion. The results indicated that IL-1β and IL-6 secretion significantly increased in monocytes overexpressing either miR-27a-3p or miR-30b-5p. When miR-27a-3p or miR-30b-5p was knocked down, IL-1β and IL-6 secretion by monocytes was significantly reduced (Figures 3A,B). Similarly, the secretion of TNF-α was significantly increased in monocytes overexpressing miR-27a-3p or miR-30b-5p. In contrast, TNF-α expression was significantly decreased when either miR-27a-3p or miR-30b-5p was knocked down (Figure 3C). The above results indicated that miR-27a-3p and miR-30b-5p can promote proinflammatory cytokine secretion by monocytes.

Monocytes were induced to differentiate by using established protocols (Zajac et al., 2013; Genin et al., 2015; Taniguchi et al., 2015). Following these protocols, monocytes were successfully induced to polarize into M1 and M1 macrophages (Figure 3D). To explore how miR-27a-3p and miR-30b-5p regulated and induced the differentiation of monocytes into macrophages, miR-27a-3p and miR-30b-5p mimics were transfected into monocytes, which were then induced to polarize into either M1 or M2 macrophages by using the previously described protocols. The cell surface expression of CD (cluster of differentiation) markers was measured to determine the differentiation ratio to assess the relationship between these two miRNAs and macrophage differentiation. Forty-eight hours after transfection with the two miRNAs and the induction of differentiation, the numbers of M0 (CD11b), M1 (CD64), and M2 (CD163, CD206) macrophages in each group were detected with flow cytometry. Generally, compared with that in the control group, the proportion of M1 macrophages was significantly higher in the experimental group. Correspondingly, compared with that in the control group, the proportion of M1 macrophages was significantly lower in the experimental group (Figures 3E–G). The above results indicated that miR-27a-3p and miR-30b-5p can promote monocytes differentiation into M1 macrophages but inhibit differentiation into M1 macrophages.

### MiR-27a-3p and miR-30b-5p downregulate the vitamin D receptor protein level

**FIGURE 4:**
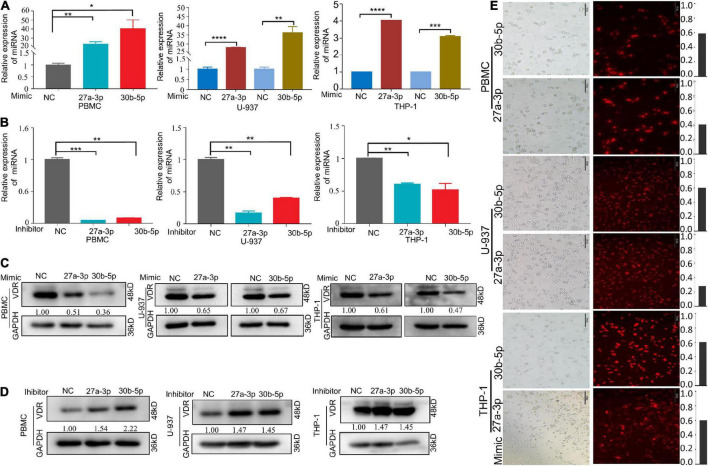
MiR-27a-3p and miR-30b-5p downregulate the protein levels of VDR in activated M1 macrophages. **(A)** The relative expression of miRNAs after transfection with miR-27a-3p and miR-30b-5p mimics in monocytes. **(B)** The relative expression of miRNAs when transfected with inhibitors. **(C)** VDR protein levels were obviously decreased when miR-27a-3p and miR-30b-5p mimics were transfected for 48 h, compared with the negative control group. **(D)** When transfected with miR-27a-3p and miR-30b-5p inhibitors, VDR protein levels were clearly upregulated compared with that in the negative control group. **(E)** The efficiency of the transfection was observed by fluorescence microscopy when miR-27a-3p and miR-30b-5p with Sulfo-Cyanine5 (Cy-5) dye were transfected into monocytes for 48 h. *P < 0.05, **P < 0.01, ***P < 0.001, ****P < 0.0001, ns = not significant.

We predicted the downstream target genes of these two miRNAs. ENCORI database prediction suggested that the VDR complex (VDR/RXR-β) might be one downstream target gene of miR-27a-3p and miR-30b-5p (Table 3). There were possible binding sites of miR-27a-3p and miR-30b-5p in VDR target genes (Table 4). Moreover, the expression of VDR measured by qRT–PCR and the level of VitD_3_ detected by ELISA were significantly reduced in the TB group compared with the control group (Supplementary Figures 2A–D), suggesting that the two factors were also significantly related to TB. To prove whether the two miRNAs could regulate the protein level of VDR, we overexpressed miR-27a-3p and miR-30b-5p in M1 macrophages. After transfection for 48 h, we observed high expression of these two miRNAs in the experimental group by qPCR (Figure 4A). Moreover, under microscopy, the transfected cells exhibited strong fluorescence signals corresponding to these two miRNAs (Figure 4E). The above results indicated the successful overexpression of the two miRNAs in monocytes.

**TABLE 3:** ENCORI miRNA-mRNA Interactions.

miRNA_ID	miRNA_name	Gene_ID	Gene_name	Gene_type	Chromosome
MIMAT0000420	hsa-miR-30b-5p	ENSG00000111424	VDR	protein_coding	chr12
MIMAT0000420	hsa-miR-30b-5p	ENSG00000111424	RXRB	protein_coding	chr6
MIMAT0000084	hsa-miR-27a-3p	ENSG00000111424	VDR	protein_coding	chr12
MIMAT0000084	hsa-miR-27a-3p	ENSG00000204231	RXRB	protein_coding	chr6

ENCORI data base prediction suggested that VDR complex (VDR/RXR-β) might be one of the downstream target genes of miR-27a-3p and miR-30b-5p.

**TABLE 4:** MicroRNA (MiRNA) target predictions based on miRanda.

miRNA	Target_gene	Score	Energy	miRNA_start	miRNA_end	CDS_UTR3_start	CDS_UTR3_end
hsa-miR-27a-3p	VDR	151	−19.36	2	19	3019	3037
hsa-miR-30b-5p	VDR	120	−8.56	2	8	1254	1275
hsa-miR-30b-5p	VDR	118	−9.47	2	20	1638	1660
hsa-miR-30b-5p	VDR	117	−12.15	3	20	1099	1119
hsa-miR-30b-5p	VDR	117	−9.67	2	21	1710	1731
hsa-miR-30b-5p	VDR	116	−9.45	2	20	210	231
hsa-miR-30b-5p	VDR	112	−5.69	3	21	2317	2338
hsa-miR-30b-5p	VDR	110	−6.6	2	11	1007	1028
hsa-miR-30b-5p	VDR	110	−14.65	3	21	1401	1425

MiRanda data base was used to predict the possible binding sites of miR-27a-3p and miR-30b-5p on VDR target gene.

Then, we measured the VDR protein levels in the experimental and control groups and found that miR-27a-3p and miR-30b-5p downregulated the protein level of VDR in the experimental group (Figure 4C). To fully elaborate these results, we knocked down either miR-27a-3p or miR-30b-5p expression in M1 macrophages. MiRNA expression and protein levels were measured separately after transfection with inhibitors. A significant decrease in miRNA expression (Figure 4B) and a significant increase in VDR protein levels (Figure 4D) were observed. However, in non-activated monocytes, neither miRNA significantly altered VDR protein expression (Supplementary Figures 3A–E). These results indicated that miR-27a-3p and miR-30b-5p are significantly involved in downregulating the protein level of VDR in activated M1 macrophages.

### MiR-27a-3p and miR-30b-5p target the 3′ untranslated region of the vitamin D receptor mRNA

**FIGURE 5:**
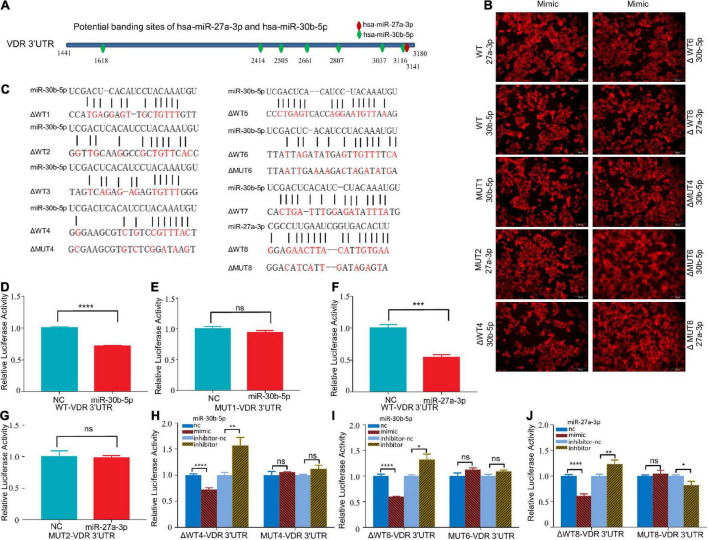
MiR-27a-3p and miR-30b-5p could bind to the 3′UTR of VDR mRNA. **(A)** The potential binding sites of miR-27a-3p and miR-30b-5p in the 3′UTR of VDR mRNA were predicted with the ENCORI database. **(B)** Red fluorescence was observed by fluorescence microscopy 48 h after transfection of HEK-293T with miR-27a-3p-Cy-5 and miR-30b-5p-Cy-5 mimics. **(C)** The sequences of the two miRNAs, wild-type plasmids and mutant-type plasmids. **(D,E)** The relative luciferase activity of the WT/MUT1-VDR-3′UTR-pmirGLO plasmid after miR-30b-5p overexpression. **(F,G)** The relative luciferase activity of the WT/MUT2- VDR-3′UTR-pmirGLO plasmids after miR-27a-3p overexpression. **(H)** The relative luciferase activity of the ΔWT4-VDR-3′UTR-pmirGLO plasmids and MUT4-VDR-3′UTR-pmirGLO plasmids after miR-30b-5p overexpression or knock down. **(I)** The relative luciferase activity of the ΔWT6-VDR-3′UTR-pmirGLO plasmids and MUT6-VDR-3′UTR-pmirGLO plasmids after miR-30b-5p overexpression or knock down. **(J)** The relative luciferase activity of the ΔWT8-VDR-3′UTR-pmirGLO plasmids and MUT8-VDR-3′UTR-pmirGLO plasmids after miR-27a-3p overexpression or knock down. *P < 0.05, **P < 0.01, ***P < 0.001, ****P < 0.0001, ns = not significant.

As previously predicted, the 3′UTR of VDR mRNA contains one binding site for miR-27a-3p and seven binding sites for miR-30b-5p (Figure 5A). We synthesized a pmirGLO reporter plasmid carrying the VDR sequence as well as a plasmid carrying mutant VDR sequences (Figure 5C). When miR-27a-3p and miR-30b-5p were overexpressed in HEK-293T cells transfected with the WT plasmid, the relative luciferase activity levels were significantly reduced (Figures 5B,D,F). When miR-30b-5p and miR-27a-3p were overexpressed in HEK-293T cells transfected with the MUT1 and MUT2 plasmids, respectively, the relative luciferase activity levels reverted to the previous levels (Figures 5B,E,G). Furthermore, we transfected the truncated mutant plasmids (ΔWT1-8) into HEK-293T cells and overexpressed either miR-27a-3p or miR-30b-5p. Both miR-27a-3p and miR-30b-5p significantly decreased the relative luciferase activities in the cells transfected with the ΔWT4, ΔWT6, and ΔWT8 plasmids. When miR-27a-3p and miR-30b-5p were overexpressed in HEK-293T cells transfected with the corresponding mutant plasmids (ΔMUT4, ΔMUT6, and ΔMUT8), the relative luciferase activities reverted to the previous levels (Figures 5B,H–J). The experiments described above indicate that there are binding sites in the 3′UTR of VDR mRNA that can be effectively bound by miR-30b-5p and miR-27a-3p.

Previous studies have suggested that miRNAs impact target genes and downregulate their expression by post-transcriptional regulation (Correia De Sousa et al., 2019). Act D can inhibit cell transcriptional activity. Therefore, we treated cells overexpressing the two miRNAs with act D (Supplementary Figures 4A–F). The VDR protein level was significantly decreased compared with that in the control group when miR-27a-3p or miR-30b-5p was overexpressed in M1 macrophages (Supplementary Figures 4G–I). These results indicated that these two miRNAs could regulate VDR protein expression at the mRNA level, namely, by reducing the stability of VDR mRNA.

### Vitamin D receptor reverses the effects of miR-27a-3p or miR-30b-5p on proinflammatory cytokine expression and macrophage differentiation

**FIGURE 6:**
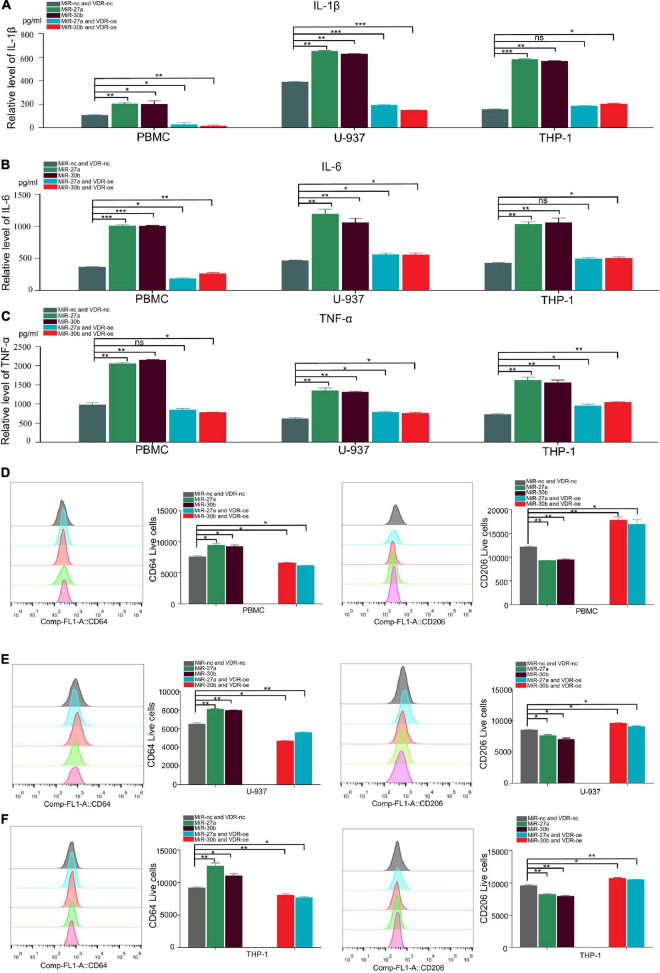
Vitamin D receptor (VDR) reverses miR-27a-3p and miR-30b-5p mediated inflammatory cytokine expression and macrophage differentiation. **(A)** MiR-27a-3p, miR-30b-5p and negative control mimics were overexpressed in activated M1 macrophages. In addition, miR-27a-3p, miR-30b-5p and negative control mimics together with VDR plasmids were transfected in another group. After 48 h, the levels of the cytokine IL-1β in the activated M1 macrophages of both groups were measured. **(B,C)** The relative levels of IL-6, TNF-α in the activated M1 macrophages of the VDR and negative control groups. **(D)** miR-27a-3p, miR-30b-5p and negative control mimics were overexpressed in PBMC M1 macrophages as a control group, while miR-27a-3p, miR-30b-5p and negative control mimics together with VDR plasmids were transfected into the VDR-OE group. The proportion of PBMCs expressing CD64 (marker of M1 macrophages) and CD206 (marker of M2 macrophages) were determined by flow cytometry. **(E)** The proportion of U-937 expressing CD64 and CD206 were determined by flow cytometry. **(F)** The proportion of THP-1 expressing CD64 and CD206 were determined by flow cytometry. *P < 0.05, **P < 0.01, ***P < 0.001, ****P < 0.0001, ns=not significant.

Previous experiments showed that miR-27a-3p and miR-30b-5p can promote proinflammatory cytokine secretion. Based on previous experiments, the VDR plasmid was overexpressed in cells first, and the cells were then transfected with miR-27a-3p or miR-30b-5p. The results showed that the proinflammatory cytokine levels in the experimental group reverted to the previous levels after VDR overexpression (Figures 6A–C). These experiments suggest that miR-27a-3p or miR-30b-5p promote the expression of proinflammatory cytokines in activated M1 macrophages by inhibiting the expression of VDR. Similarly, based on previous experiments, we transfected either miR-27a-3p or miR-30b-5p into monocytes after VDR overexpression. The results showed that macrophage differentiation in the experimental group reverted to the previous levels after VDR overexpression (Figures 6D–F). Gating strategy for flow cytometry assay is shown in Supplementary Figure 5. These experiments suggest that miR-27a-3p and miR-30b-5p induced M1 macrophage differentiation and inhibited M2 macrophage differentiation by inhibiting VDR.

### By repressing vitamin D receptor protein expression, miR-27a-3p and miR-30b-5p activate the NF-kB signaling pathway and subsequently promote proinflammatory cytokine secretion and macrophage differentiation

**FIGURE 7:**
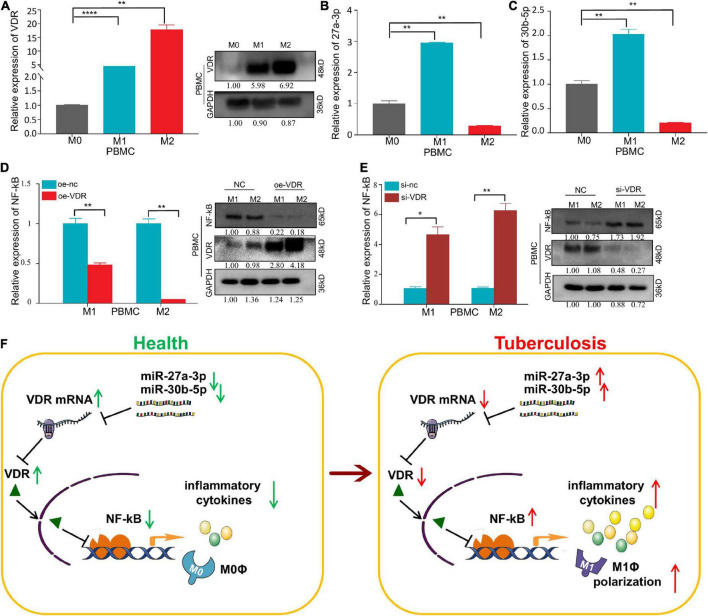
An opposite trend of VDR and miRNA expression has been observed and VDR can inhibit the expression of NF-kB p65 in macrophages. **(A)** The relative expression of VDR in PBMC–derived M0, M1, and M2 macrophages significantly increased successively, as determined by qRT–PCR and Western blot assay. **(B)** The relative expression of miR-27a-3p in PBMC-derived M0, M1, and M2 macrophages. Compared with M0 macrophages, the relative expression of miR-27a-3p in M1 macrophages was significantly increased, but it was significantly decreased in M2 macrophages. **(C)** Compared with M0 macrophages, the relative expression of miR-30b-5p in M1 macrophages was also significantly increased, but it also significantly decreased in M2 macrophages. **(D)** After overexpression of VDR in PBMC-derived M1 and M2 macrophages, the expression of NF-kB p65 was measured by qRT–PCR and Western blot assay. Compared with the control group, the expression of NF-kB p65 was significantly decreased in M1 and M2 macrophages, which was opposite to the VDR expression trend. **(E)** After knocking down VDR expression in PBMC-derived M1 and M2 macrophages, the expression of NF-kB p65 was measured by qRT–PCR and Western blot assay. Compared with the control group, the expression of NF-kB p65 was significantly increased in M1 and M2 macrophages, which was also opposite to the VDR expression trend. **(F)** Mechanism chart. MiR-27a-3p and miR-30b-5p inhibit the VDR protein levels by targeting VDR mRNA. The decrease of VDR activated NF-kB signaling pathway and increased the secretion of proinflammatory cytokines and simultaneously, induced monocytes differentiation into M1 macrophages. *P < 0.05, **P < 0.01, ***P < 0.001, ****P < 0.0001, ns = not significant.

VDR expression was highly expressed in both M1 and M2 macrophages, and expression in the latter was higher than that in the former (Figure 7A). MiR-27a-3p and miR-30b-5p expression are highly expressed in M1 macrophages, but lowly expressed in M2 macrophages (Figures 7B,C). We also found that there was a protein–protein interaction between VDR and NF-kB p65 and that VDR significantly inhibited NF-kB p65 protein expression (Figures 7D,E). Complete strips images of western blot are shown in Supplementary Figures 6–8. This finding suggests that one of the ways in which miRNAs regulate proinflammatory cytokine secretion and macrophage differentiation is by decreasing VDR protein levels, which at the same time activates the NF-kB signaling pathway and finally promotes the progression of TB.

Taken together, the above results indicate that miR-27a-3p and miR-30b-5p play vital roles in promoting the progression of TB. On the one hand, by targeting and downregulating VDR mRNA expression, these two miRNAs upregulate the NF-kB signaling pathway and lead to the increased secretion of proinflammatory cytokines, thus promoting the progression of TB. On the other hand, by inducing the differentiation of classically activated M1 macrophages, these two miRNAs mediate antibacterial defenses in patients with TB (Figure 7F).

## Discussion

MicroRNAs regulate biological processes by targeting genes, thereby influencing the development of diseases (Huang et al., 2021). MiRNAs are expected to become new biomarkers for diagnosis, therapy prognosis prediction and treatment in breast cancer (Bertoli et al., 2015). Studies have shown that miRNAs target VDR in breast carcinoma and liver fibrosis and then affect disease progression (Liu X. et al., 2018; He et al., 2021), but whether these miRNAs are differentially expressed in TB is still unclear. It has been reported that miRNAs such as miR-92a-3p and miR-155 are highly expressed in TB and have an impact on the occurrence of the disease (Wu et al., 2012; Wang et al., 2018), but much work still remains to be done to clarify the mechanisms underlying this phenomenon. Some studies have shown that miRNAs play a vital role in the regulation of inflammatory cytokines (Zumkehr et al., 2018; Ban et al., 2020; Zhang et al., 2021) and that increased proinflammatory cytokine secretion by PBMCs or macrophages stimulated by other known factors can promote wound healing (Xie et al., 2021). Therefore, we wanted to screen miRNAs and investigate whether they meaningfully affect disease progression through known inflammatory pathways in TB. To determine which miRNAs can modulate the pathogenesis of TB and how they exert this function, the screened miRNAs should meet two conditions: first, they must be differentially expressed in TB. Second, they should have some target gene with a close connection to TB.

Macrophages have a variety of physiological functions, including tissue repair, osteoclasts, antigen and antibody uptake, phagocytosis, antimicrobial effects, and antigen presentation, among others (Chistiakov et al., 2018). Macrophages have plasticity and phenotypic heterogeneity, can be induced to differentiate into M1 classical and M2 alternative activation forms, and play important roles in promoting and inhibiting inflammation in chronic inflammatory diseases (Parisi et al., 2018). Generally, M1 macrophages appear in the earlier stage of inflammation and can kill and remove pathogens and damaged cells, while M2 macrophages promote the regeneration and homeostasis of damaged tissues in the later stage (Atri et al., 2018; Zhou et al., 2019). Therefore, remodeling macrophage polarization to regulate the inflammatory process is considered a new prospective approach to treat inflammatory diseases. Many determinant factors can stimulate and induce macrophage polarization and shape macrophage phenotype and function, including metabolism and microbial metabolites, cellular metabolites, damaged cells, activated lymphocytes, miRNAs, inflammatory cytokines, interferon regulator factors, and epigenetic factors. Therefore, we chose to detect the differential expression of miRNAs in macrophages for the study of TB.

Vitamin D receptor pathway activation shows certain anti-inflammatory effects. Research shows that the activation of VDR signaling represses inflammation and transforms Kupffer cells from a proinflammatory to an anti-inflammatory state (Dong et al., 2020). Another study suggested that VDR signaling induces macrophage differentiation and skews myelofibrosis (Wakahashi et al., 2019). The NF-kB pathway is considered to play a vital role in regulating inflammation. A study showed that the activation of NF-kB promotes M1 macrophage polarization and proinflammatory cytokine secretion, thereby promoting inflammatory disease progression (Lv et al., 2020). Research shows that VDR blocks NF-kB activation by decreasing the levels of the IKK complex (Chen et al., 2013), which was confirmed in our study. Many miRNAs target VDR in a specific manner. Through bioinformatics prediction and validation, we identified VDR as the target gene of the two miRNAs in this study. We also found that VDR could reverse the progression of M1 macrophage polarization and proinflammatory cytokine secretion induced by miRNAs.

Therefore, in our study, we first screened miRNAs differentially expressed in TB and then investigated the function and mechanism of the identified miRNAs. Through comparisons of TB patients and controls, high-throughput sequencing and qRT–PCR were used to screen miR-27a-3p and miR-30b-5p, which were expressed at significantly higher levels in the TB group. Western blot experiments confirmed that the two miRNAs significantly inhibited VDR protein expression in differentiated macrophages but not in undifferentiated macrophages. Further experiments showed that miR-27a-3p or miR-30b-5p could significantly downregulate VDR mRNA expression in activated M1 macrophages. The miR-27a-3p and miR-30b-5p binding sites in the 3′UTR of VDR mRNA were verified by dual-luciferase assays. More specifically, ELISA and flow cytometry showed that the two miRNAs could promote M1 macrophage polarization and proinflammatory cytokine secretion. Moreover, after VDR overexpression, macrophage differentiation and proinflammatory cytokine secretion were reversed to a certain extent. The above results suggest that miR-27a-3p and miR-30b-5p induce M1 macrophage differentiation and upregulate the expression of proinflammatory cytokines by targeting VDR. Moreover, we found that the roles of these miRNAs in TB were previously reported in two different studies (Xin et al., 2016; Liu F. et al., 2018). These two studies clearly illustrated the important regulatory roles of these two miRNAs in TB. However, the relationship of miR-27a-3p and miR-30b-5p with VDR protein levels was studied here for the first time.

In conclusion, this study identified miR-27a-3p and miR-30b-5p in TB patients. Both miRNAs target VDR mRNA, promote proinflammatory cytokine expression, and induce the polarization of monocytes into M1 macrophages while inhibiting the polarization of monocytes into M2 macrophages. Therefore, we conclude that the high expression of these two miRNAs is credibly related to the progression of TB.

## Data availability statement

The datasets presented in this study are deposited in the NCBI GEO repository, accession number GSE207224, available at https://www.ncbi.nlm.nih.gov/geo/query/acc.cgi?acc=GSE20 7244.

## Ethics statement

The studies involving human participants were reviewed and approved by Xinqiao Hospital Ethics Committee. The patients/participants provided their written informed consent to participate in this study.

## Author contributions

All authors listed have made a substantial, direct, and intellectual contribution to the work, and approved it for publication.

## References

[B1] Atri C. Guerfali F. Laouini D. (2018). Role of human macrophage polarization in inflammation during infectious diseases. *Int. J. Mol. Sci.* 19:1801. 10.3390/ijms19061801 PMC6032107 29921749

[B2] Ayelign B. Workneh M. Molla M. D. Dessie G. (2020). Role of vitamin-D supplementation in TB/HIV co-infected patients. *Infect. Drug Resist.* 13 111–118. 10.2147/IDR.S228336 32021325 PMC6959508

[B3] Baeke F. Takiishi T. Korf H. Gysemans C. Mathieu C. (2010). Vitamin D: Modulator of the immune system. *Curr. Opin. Pharmacol.* 10 482–496. 20427238 10.1016/j.coph.2010.04.001

[B4] Ban E. Jeong S. Park M. Kwon H. Park J. Song E. J. (2020). Accelerated wound healing in diabetic mice by miRNA-497 and its anti-inflammatory activity. *Biomed. Pharmacother.* 121:109613. 10.1016/j.biopha.2019.109613 31707336

[B5] Barry S. E. Ellis M. Yang Y. Guan G. Wang X. Britton W. J. (2018). Identification of a plasma microRNA profile in untreated pulmonary tuberculosis patients that is modulated by anti-mycobacterial therapy. *J. Infect.* 77 341–348. 10.1016/j.jinf.2018.03.006 29746939

[B6] Bartel D. P. (2004). Micrornas: Genomics, biogenesis, mechanism, and function. *Cell* 116 281–297. 14744438 10.1016/s0092-8674(04)00045-5

[B7] Bekele A. Gebreselassie N. Ashenafi S. Kassa E. Aseffa G. Amogne W. (2018). Daily adjunctive therapy with vitamin D3 and phenylbutyrate supports clinical recovery from pulmonary tuberculosis: A randomized controlled trial in Ethiopia. *J. Intern. Med.* 284 292–306. 10.1111/joim.12767 29696707 PMC6202271

[B8] Bertoli G. Cava C. Castiglioni I. (2015). Micrornas: New biomarkers for diagnosis, prognosis, therapy prediction and therapeutic tools for breast cancer. *Theranostics* 5 1122–1143. 26199650 10.7150/thno.11543 PMC4508501

[B9] Böyum A. Scand J. (1968). Isolation of leucocytes from human blood. A two-phase system for removal of red cells with methylcellulose as erythrocyte-aggregating agent. *Scand. J. Clin. Lab. Invest. Suppl.* 97 9–29. 4974753

[B10] Chen Y. Zhang J. Ge X. Du J. Deb D. K. Li Y. C. (2013). Vitamin D receptor inhibits nuclear factor κB activation by interacting with IκB kinase β protein. *J. Biol. Chem.* 288 19450–19458. 23671281 10.1074/jbc.M113.467670 PMC3707648

[B11] Chistiakov D. A. Myasoedova V. A. Revin V. V. Orekhov A. N. Bobryshev Y. V. (2018). The impact of interferon-regulatory factors to macrophage differentiation and polarization into M1 and M2. *Immunobiology* 223 101–111. 10.1016/j.imbio.2017.10.005 29032836

[B12] Correia De Sousa M. Gjorgjieva M. Dolicka D. Sobolewski C. Foti M. (2019). Deciphering mirnas’ action through mirna editing. *Int. J. Mol. Sci.* 20:6249. 10.3390/ijms20246249 31835747 PMC6941098

[B13] Cui X. Pertile R. Eyles D. W. (2018). The vitamin D receptor (VDR) binds to the nuclear matrix via its hinge domain: A potential mechanism for the reduction in VDR mediated transcription in mitotic cells. *Mol. Cell. Endocrinol.* 472 18–25. 10.1016/j.mce.2017.11.015 29183808

[B14] Dai S. Wen Y. Luo P. Ma L. Liu Y. Ai J. (2022). Therapeutic implications of exosomes in the treatment of radiation injury. *Burns Trauma* 10:tkab043. 10.1093/burnst/tkab043 35071650 PMC8778593

[B15] Daley C. L. (2019). The global fight against tuberculosis. *Thorac. Surg. Clin.* 29 19–25. 30454918 10.1016/j.thorsurg.2018.09.010

[B16] Dong B. Zhou Y. Wang W. Scott J. Kim K. Sun Z. (2020). Vitamin D receptor activation in liver macrophages ameliorates hepatic inflammation, steatosis, and insulin resistance in mice. *Hepatology* 71 1559–1574. 10.1002/hep.30937 31506976

[B17] Etna M. P. Sinigaglia A. Grassi A. Giacomini E. Romagnoli A. Pardini M. (2018). *Mycobacterium tuberculosis*-induced miR-155 subverts autophagy by targeting Atg3 in human dendritic cells. *PLoS Pathog.* 14:e1006790. 10.1371/journal.ppat.1006790 29300789 PMC5771628

[B18] Genin M. Clement F. Fattaccioli A. Raes M. Michiels C. (2015). M1 and M2 macrophages derived from Thp-1 cells differentially modulate the response of cancer cells to etoposide. *BMC Cancer* 15:577. 10.1186/s12885-015-1546-9 26253167 PMC4545815

[B19] Goldberg M. F. Saini N. K. Porcelli S. A. (2014). Evasion of innate and adaptive immunity by *Mycobacterium tuberculosis*. *Microbiol. Spectr.* 2. 10.1128/microbiolspec.MGM2-0005-2013 26104343

[B20] Gong Z. Kuang Z. Li H. Li C. Ali M. K. Huang F. (2019). Regulation of host cell pyroptosis and cytokines production by *Mycobacterium tuberculosis* effector PPE60 requires LUBAC mediated Nf-κB signaling. *Cell. Immunol.* 335 41–50. 10.1016/j.cellimm.2018.10.009 30415762

[B21] Harris R. Ukayiofo E. (1969). Rapid preparation of lymphocytes for tissue-typing. *Lancet* 2:327. 10.1016/s0140-6736(69)90096-8 4184246

[B22] He W. Ni W. Zhao L. Wang X. Liu L. Fan Z. (2021). Microrna-125a/VDR axis impaired autophagic flux and contributed to fibrosis in a Ccl4-induced mouse model and patients with liver cirrhosis. *Life Sci.* 264:118666. 10.1016/j.lfs.2020.118666 33130085

[B23] Heikkinen S. Väisänen S. Pehkonen P. Seuter S. Benes V. Carlberg C. (2011). Nuclear hormone 1α,25-dihydroxyvitamin D3 elicits a genome-wide shift in the locations of VDR chromatin occupancy. *Nucleic Acids Res.* 39 9181–9193. 10.1093/nar/gkr654 21846776 PMC3241659

[B24] Huang H. Lin Y. Cui S. Huang Y. Tang Y. (2021). miRTarBase update 2022an informative resource forexperimentally validated miRNA–target interactions. *Nucleic Acids Res.* 50 222–230. 10.1093/nar/gkab1079 PMC8728135 34850920

[B25] Jagielski T. Minias A. van Ingen J. Rastogi N. Brzostek A. Żaczek A. (2016). Methodological and clinical aspects of the molecular epidemiology of *Mycobacterium tuberculosis* and other *Mycobacteria*. *Clin. Microbiol. Rev.* 29 239–290. 26912567 10.1128/CMR.00055-15 PMC4786889

[B26] Jiang H. Sun J. Chen Y. Chen Z. Wang L. Gao W. (2018). Landscape of the genome and host cell response of *Mycobacterium shigaense* reveals pathogenic features. *Emerg. Microbes Infect.* 7:112. 10.1038/s41426-018-0116-z 29934568 PMC6015043

[B27] Jimenez-Sousa M. A. Martinez I. Medrano L. M. Fernandez-Rodriguez A. Resino S. (2018). Vitamin D in human immunodeficiency virus infection: Influence on immunity and disease. *Front. Immunol.* 9:458. 10.3389/fimmu.2018.00458 29593721 PMC5857570

[B28] Joshi L. Ponnana M. Penmetsa S. R. Nallari P. Valluri V. Gaddam S. (2014). Serum vitamin D levels and VDR polymorphisms (BsmI and FokI) in patients and their household contacts susceptible to tuberculosis. *Scand. J. Immunol.* 79 113–119. 10.1111/sji.12127 24219580

[B29] Liu F. Chen J. Wang P. Li H. Zhou Y. Liu H. (2018). Microrna-27a controls the intracellular survival of *Mycobacterium tuberculosis* by regulating calcium-associated autophagy. *Nat. Commun.* 9:4295. 10.1038/s41467-018-06836-4 30327467 PMC6191460

[B30] Liu P. T. Stenger S. Li H. Wenzel L. Tan B. H. Krutzik S. R. (2006). Toll-like receptor triggering of a vitamin D-mediated human antimicrobial response. *Science* 311 1770–1773. 10.1126/science.1123933 16497887

[B31] Liu X. Bi L. Wang Q. Wen M. Li C. Ren Y. (2018). miR-1204 targets VDR to promotes epithelial-mesenchymal transition and metastasis in breast cancer. *Oncogene* 37 3426–3439. 10.1038/s41388-018-0215-2 29555976

[B32] Lv L. Feng Y. Wu M. Wang B. Li Z. Zhong X. (2020). Exosomal miRNA-19b-3p of tubular epithelial cells promotes M1 macrophage activation in kidney injury. *Cell Death Differ.* 27 210–226. 10.1038/s41418-019-0349-y 31097789 PMC7206053

[B33] McNerney R. Maeurer M. Abubakar I. Marais B. Mchugh T. D. Ford N. (2012). Tuberculosis diagnostics and biomarkers: Needs, challenges, recent advances, and opportunities. *J. Infect. Dis.* 205(Suppl. 2) S147–S158. 22496353 10.1093/infdis/jir860

[B34] Mohr A. M. Mott J. L. (2015). Overview of microrna biology. *Semin. Liver Dis.* 35 3–11. 25632930 10.1055/s-0034-1397344 PMC4797991

[B35] Nair S. Ramaswamy P. A. Ghosh S. Joshi D. C. Pathak N. Siddiqui I. (2009). The PPE18 of *Mycobacterium tuberculosis* interacts with TLR2 and activates IL-10 induction in macrophage. *J. Immunol.* 183 6269–6281. 10.4049/jimmunol.0901367 19880448

[B36] Nnoaham K. E. Clarke A. (2008). Low serum vitamin D levels and tuberculosis: A systematic review and meta-analysis. *Int. J. Epidemiol.* 37 113–119. 18245055 10.1093/ije/dym247

[B37] Panda S. Tiwari A. Luthra K. Sharma S. K. Singh A. (2019). Status of vitamin D and the associated host factors in pulmonary tuberculosis patients and their household contacts: A cross sectional study. *J. Steroid Biochem. Mol. Biol.* 193:105419. 10.1016/j.jsbmb.2019.105419 31255688

[B38] Parisi L. Gini E. Baci D. Tremolati M. Fanuli M. Bassani B. (2018). Macrophage polarization in chronic inflammatory diseases: Killers or builders? *J. Immunol. Res.* 2018:8917804. 10.1155/2018/8917804 PMC5821995 29507865

[B39] Pieters J. (2008). *Mycobacterium tuberculosis* and the macrophage: Maintaining a balance. *Cell Host Microbe* 3 399–407. 10.1016/j.chom.2008.05.006 18541216

[B40] Ravan P. Nejad Sattari T. Siadat S. D. Vaziri F. (2019). Evaluation of the expression of cytokines and chemokines in macrophages in response to rifampin-monoresistant *Mycobacterium tuberculosis* and H37Rv strain. *Cytokine* 115 127–134. 10.1016/j.cyto.2018.12.004 30594437

[B41] Ren H. Zhao F. Zhang Q. Huang X. Wang Z. (2022). Autophagy and skin wound healing. *Burns Trauma* 10:tkac003. 10.1093/burnst/tkac003 PMC8847901 35187180

[B42] Rupaimoole R. Slack F. J. (2017). MicroRNA therapeutics: Towards a new era for the management of cancer and other diseases. *Nat. Rev. Drug Discov.* 16 203–222. 10.1038/nrd.2016.246 28209991

[B43] Selvaraj P. Prabhu Anand S. Harishankar M. Alagarasu K. (2009). Plasma 1,25 dihydroxy vitamin D3 level and expression of vitamin D receptor and cathelicidin in pulmonary tuberculosis. *J. Clin. Immunol.* 29 470–478. 10.1007/s10875-009-9277-9 19219539

[B44] Sinha P. Hochberg N. S. (2019). Crystal ball: The yesterday and tomorrow of tuberculosis. *Environ. Microbiol. Rep.* 11 41–44. 10.1111/1758-2229.12726 30585431

[B45] Taniguchi K. Hikiji H. Okinaga T. Hashidate-Yoshida T. Shindou H. Ariyoshi W. (2015). Essential role of lysophosphatidylcholine acyltransferase 3 in the induction of macrophage polarization in PMA-treated U937 cells. *J. Cell. Biochem.* 116 2840–2848. 10.1002/jcb.25230 25994902

[B46] Wakahashi K. Minagawa K. Kawano Y. Kawano H. Suzuki T. Ishii S. (2019). Vitamin D receptor–mediated skewed differentiation of macrophages initiates myelofibrosis and subsequent osteosclerosis. *Blood* 133 1619–1629. 10.1182/blood-2018-09-876615 30718230

[B47] Wallis R. S. Zumla A. (2016). Vitamin D as adjunctive host-directed therapy in tuberculosis: A systematic review. *Open Forum Infect. Dis.* 3:ofw151. 10.1093/ofid/ofw151 PMC5084719 27800526

[B48] Wang C. Yang S. Liu C. Jiang T. Chen Z. Tu H. (2018). Screening and identification of four serum mirnas as novel potential biomarkers for cured pulmonary tuberculosis. *Tuberculosis* 108 26–34. 10.1016/j.tube.2017.08.010 29523324

[B49] White J. H. (2012). Vitamin D metabolism and signaling in the immune system. *Rev. Endocr. Metab. Disord.* 13 21–29. 21845364 10.1007/s11154-011-9195-z

[B50] World Health Organization [WHO] (2019). *Global TB report.* Geneva: World Health Organization.

[B51] Wu J. Lu C. Diao N. Zhang S. Wang S. Wang F. (2012). Analysis of microrna expression profiling identifies miR-155 and miR-155* as potential diagnostic markers for active tuberculosis: A preliminary study. *Hum. Immunol.* 73 31–37. 10.1016/j.humimm.2011.10.003 22037148

[B52] Wu L. Deng H. Zheng Y. Mansjö M. Zheng X. Hu Y. (2015). An association study of Nramp1, VDR, MBL and their interaction with the susceptibility to tuberculosis in a Chinese population. *Int. J. Infect. Dis.* 38 129–135. 10.1016/j.ijid.2015.08.003 26261060

[B53] Xie W. Zhou X. Hu W. Chu Z. Ruan Q. Zhang H. (2021). Pterostilbene accelerates wound healing by modulating diabetes-induced estrogen receptor β suppression in hematopoietic stem cells. *Burns Trauma* 9:tkaa045. 10.1093/burnst/tkaa045 33654697 PMC7901710

[B54] Xin H. Yang Y. Liu J. Li X. Li M. Feng B. (2016). Association between tuberculosis and circulating microRNA hsa-let-7b and hsa-miR-30b: A pilot study in a Chinese population. *Tuberculosis* 99 63–69. 10.1016/j.tube.2016.04.005 27450007

[B55] Zajac E. Schweighofer B. Kupriyanova T. A. Juncker-Jensen A. Minder P. Quigley J. P. (2013). Angiogenic capacity of M1- and M2-polarized macrophages is determined by the levels of Timp-1 complexed with their secreted prommp-9. *Blood* 122 4054–4067. 10.1182/blood-2013-05-501494 24174628 PMC3862278

[B56] Zhang P. Zou B. Liou Y. Huang C. (2021). The pathogenesis and diagnosis of sepsis post burn injury. *Burns Trauma* 9:tkaa047. 10.1093/burnst/tkaa047 PMC7901709 33654698

[B57] Zhou X. Li W. Wang S. Zhang P. Wang Q. Xiao J. (2019). YAP aggravates inflammatory bowel disease by regulating M1/M2 macrophage polarization and gut microbial homeostasis. *Cell Rep.* 27 1176–1189.e5. 10.1016/j.celrep.2019.03.028 31018132

[B58] Zumkehr J. Rodriguez-Ortiz C. J. Medeiros R. Kitazawa M. (2018). Inflammatory cytokine, Il-1β, regulates glial glutamate transporter *via* microRNA-181a *in vitro*. *J. Alzheimers Dis.* 63 965–975. 10.3233/JAD-170828 29710703 PMC7325598

