# Supplementary material for: MiR-27a-3p and miR-30b-5p inhibited-vitamin D receptor involved in the progression of tuberculosis
Source: Front Microbiol. 2022 Oct 11;13:1020542. doi: 10.3389/fmicb.2022.1020542 (PMC9593098; doi:10.3389/fmicb.2022.1020542)
Supplement: Supplementary file 1 [file Data_Sheet_1.docx]

**Supplementary figure 1 MiRNAs high-throughput sequencing. ab** Forty-seven miRNAs were differentially expressed (p<0.05) between the TB group and the control group. Among them, 23 miRNAs had a significant fold-change in expression (|Log_2_(FC)|>1). Among these miRNAs, 8 miRNAs were upregulated, and 15 miRNAs were downregulated. **c** Principal component analysis: 9 of 10 samples could be clustered together in a similar dimension.

**Supplementary figure 2 VDR expresses lower in the tuberculosis group compared with control group. a** The relative expression of VDR in TB group was significantly lower than control group. **b** Similarly, VitD_3_ concentration in plasma in TB group was significantly lower than control group. **c** Further, the concentration of VitD_3_ was divided into four levels: deficiency, insufficient, sufficient and intoxication. In sufficient level, VitD_3_ concentration in TB group was significantly lower than control group. **d** And TB group had a smaller percentage in sufficient level and a larger percentage in insufficient level compared with control group.

**Supplementary figure3 MiR-27a-3p and miR-30b-5p could hardly regulated VDR expression in nonactivated U-937 and THP-1. a** The transfection efficiency after transfected with miR-27a-3p-Cy-5 and miR-30b-5p-Cy-5 mimics 48h later by fluorescence microscopy.**bc** The transfection efficiency of U-937 and THP-1 after transfected miRNAs 48h later by qRT-PCR. **de** VDR protein levels were slightly decreased after transfection of two miRNAs 48h later in U-937 and THP-1.

**Supplementary figure 4 MiR-27a-3p and miR-30b-5p could down regulate VDR at mRNA level in activated M1 macrophages. abc**M1 macrophages were transfected with miR-27a-3p, miR-30b-5p and negative control mimics, respectively. And the expressions of VDR were detected 48 h later. **def** The relative expression of miR-27a-3p and miR-30b-5p in activated M1 macrophages after transfected for 36h (Actinomycin D treated), 39h, 42h, 45h. **ghi** M1 macrophages were transfected with miR-27a-3p, miR-30b-5p and negative control mimics, respectively, and cultured for 36h. Then cells were treated with Actinomycin D at 0h, 3h, 6h and 9h, and total RNA was extracted to detect the relative expression of VDR mRNA by qRT-PCR.

**Supplementary figure 5 Flow cytometry assay gating strategy.**

**Supplementary figure 6 Western blot membrane image of Figure 4.**

**Supplementary figure 7 Western blot membrane image of Figure 7.**

**Supplementary figure 8 Western blot membrane image of Supplementary Figure 3.**
