# Supplementary figures and images for: MiR-27a-3p and miR-30b-5p inhibited-vitamin D receptor involved in the progression of tuberculosis
Source: Front Microbiol. 2022 Oct 11;13:1020542. doi: 10.3389/fmicb.2022.1020542 (PMC9593098; doi:10.3389/fmicb.2022.1020542)

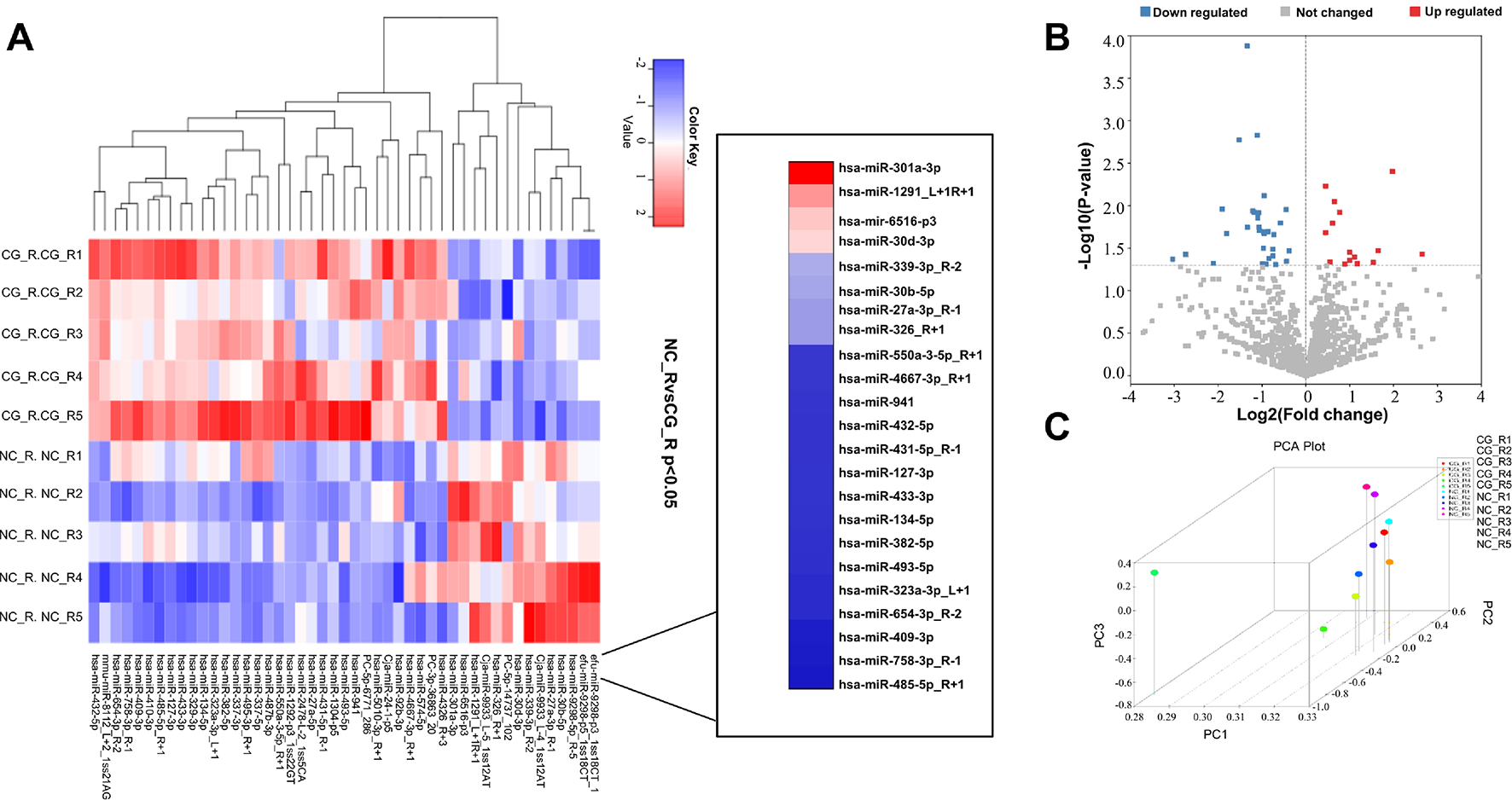

Supplement: Supplementary file 2 [file Image_1.TIFF]

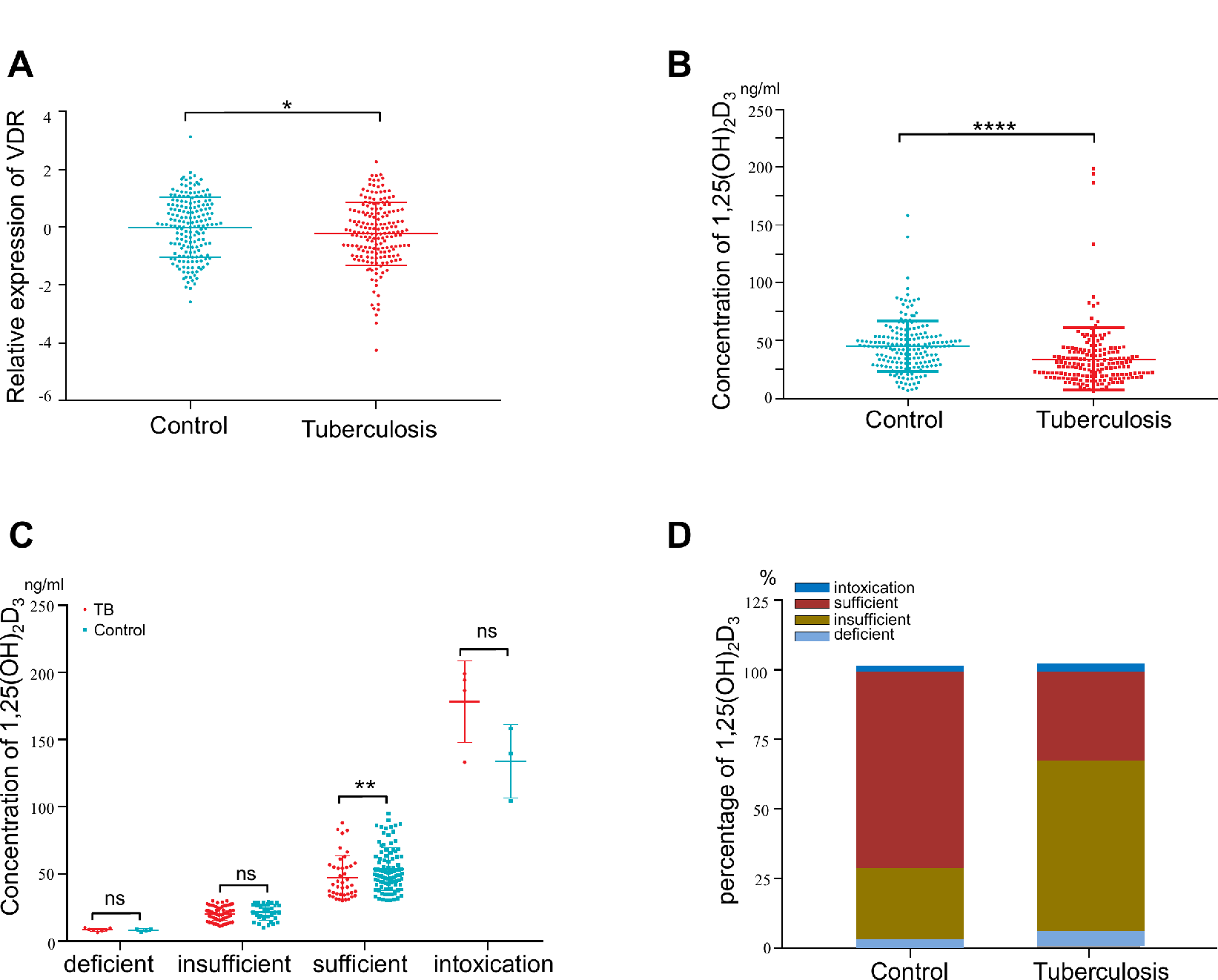

Supplement: Supplementary file 3 [file Image_2.TIFF]

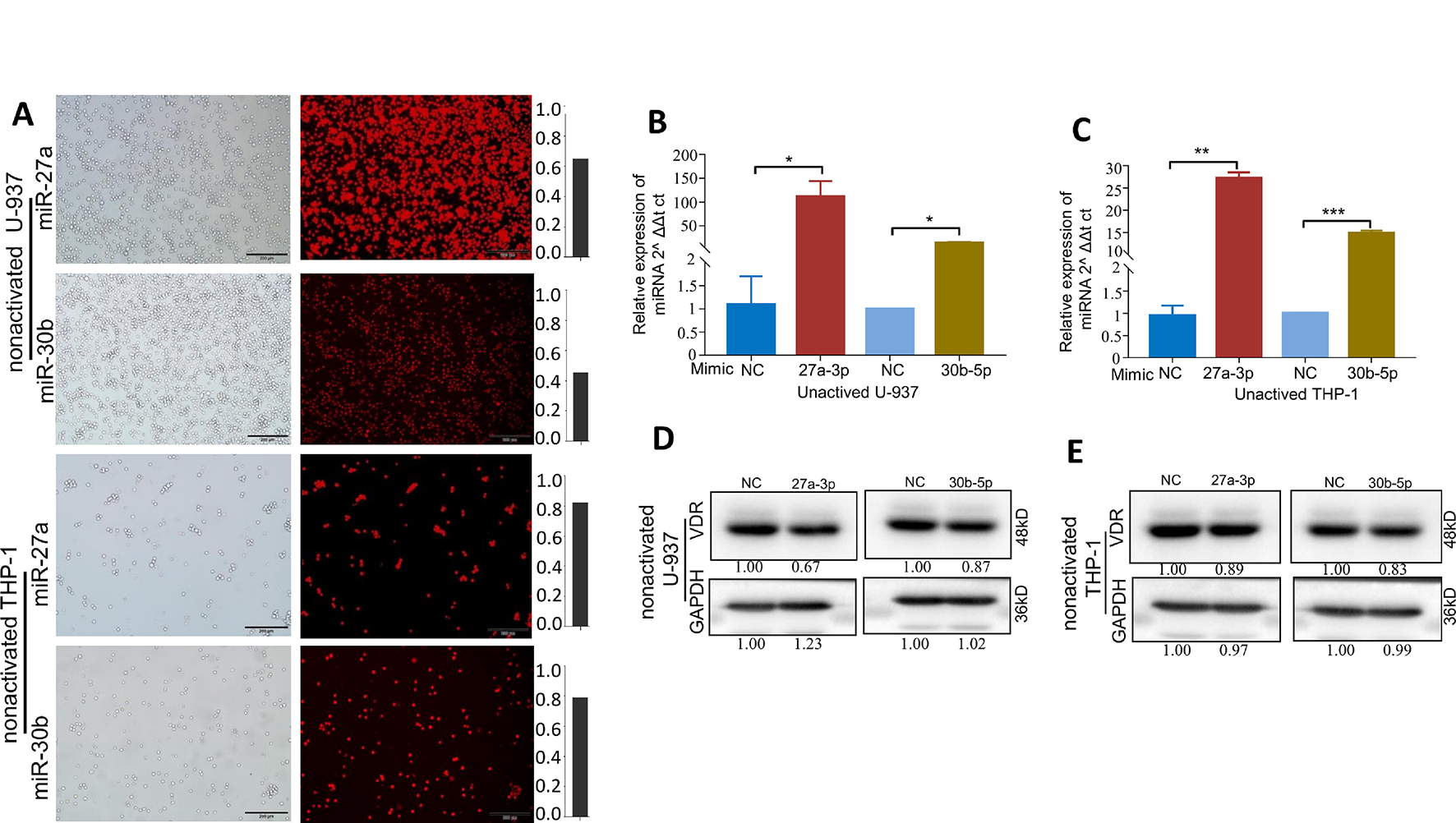

Supplement: Supplementary file 4 [file Image_3.TIFF]

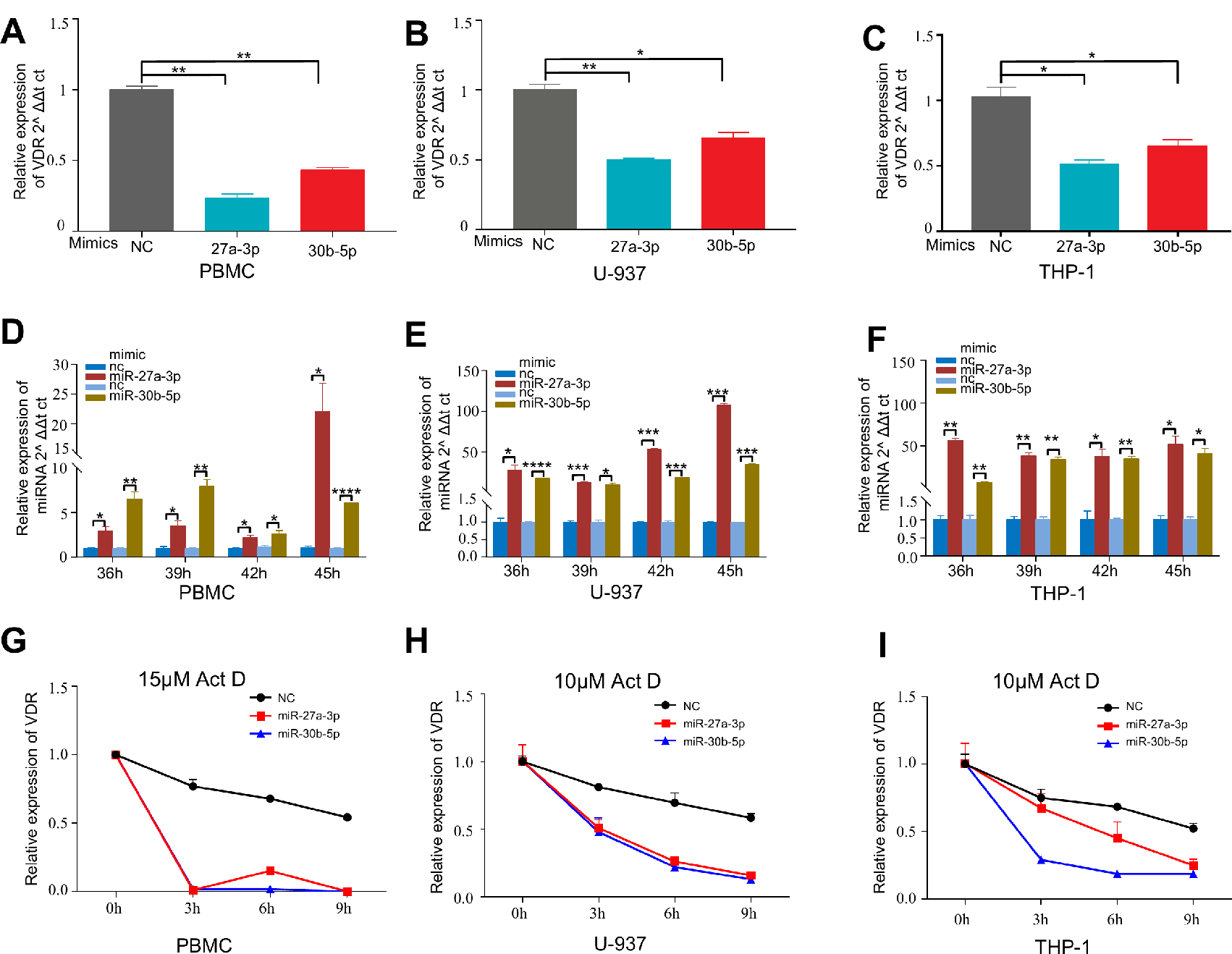

Supplement: Supplementary file 5 [file Image_4.TIFF]

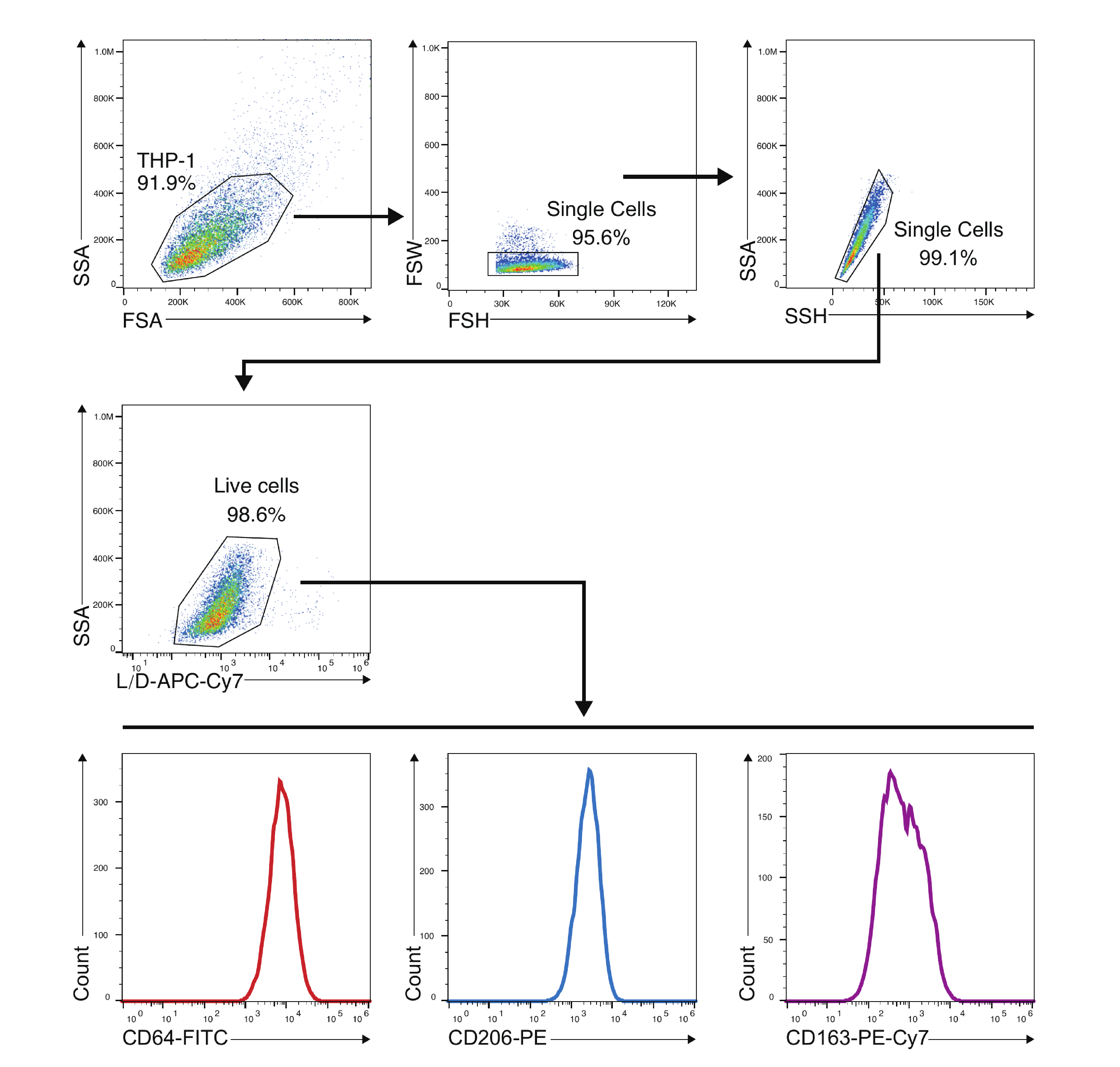

Supplement: Supplementary file 6 [file Image_5.TIFF]

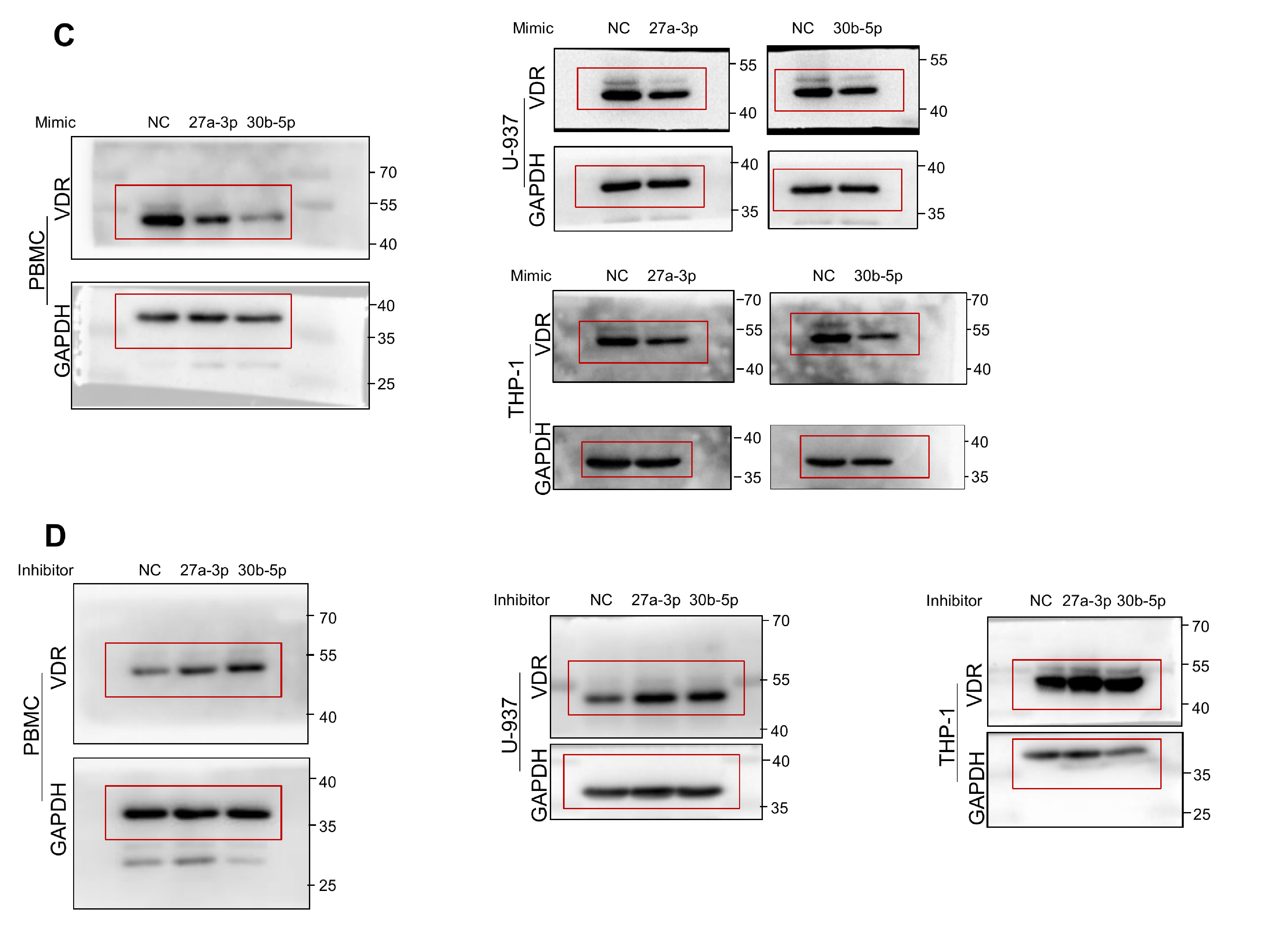

Supplement: Supplementary file 7 [file Image_6.TIFF]

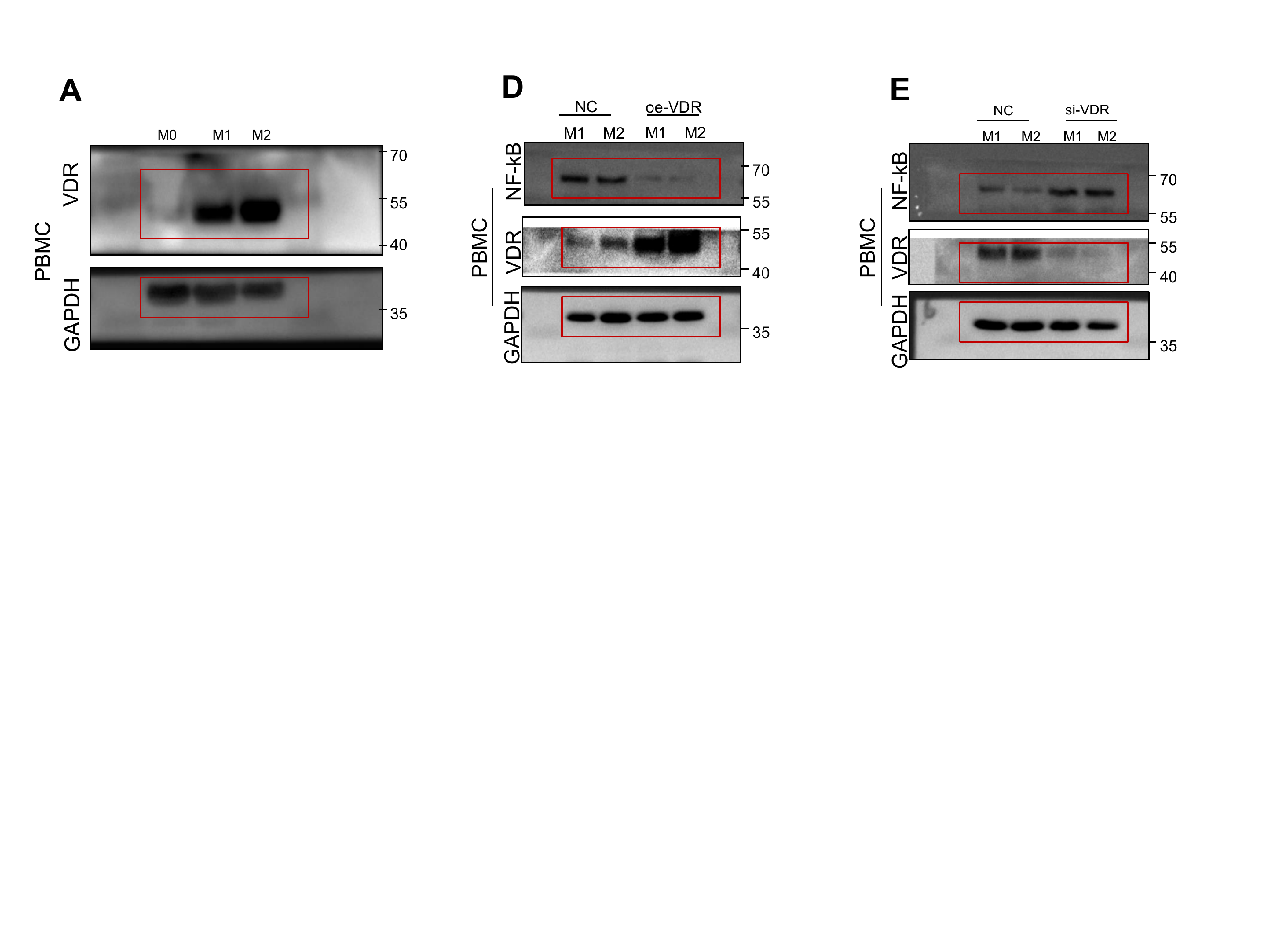

Supplement: Supplementary file 8 [file Image_7.TIFF]

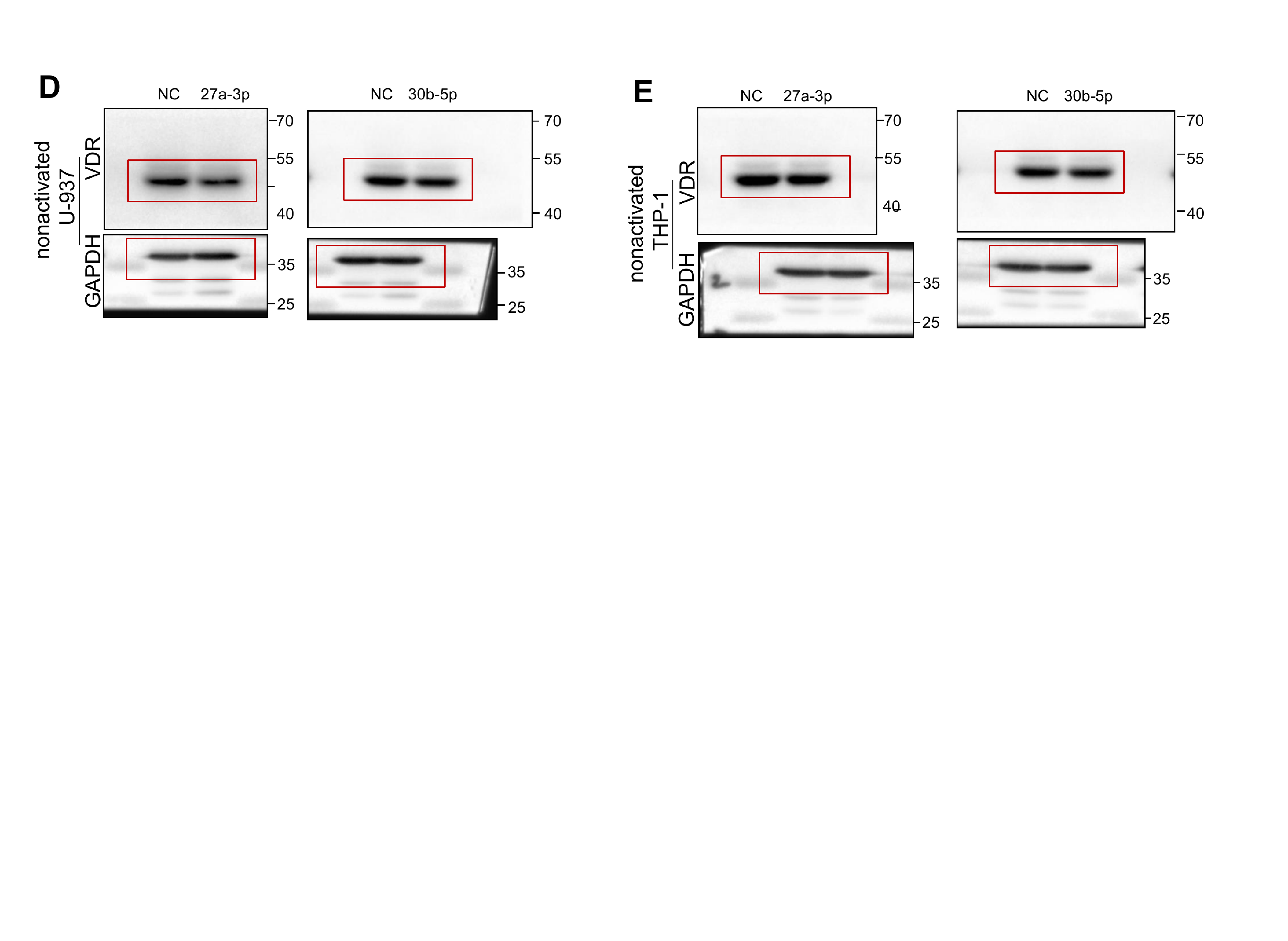

Supplement: Supplementary file 9 [file Image_8.TIFF]
